# G Protein-Coupled Receptors in Taste Physiology and Pharmacology

**DOI:** 10.3389/fphar.2020.587664

**Published:** 2020-11-30

**Authors:** Raise Ahmad, Julie E. Dalziel

**Affiliations:** Food Nutrition and Health Team, Food and Bio-based Products Group, AgResearch, Palmerston North, New Zealand

**Keywords:** taste receptor, taste bud, flavor, taste ligand, signal transduction, gustducin, peptide

## Abstract

Heterotrimeric G protein-coupled receptors (GPCRs) comprise the largest receptor family in mammals and are responsible for the regulation of most physiological functions. Besides mediating the sensory modalities of olfaction and vision, GPCRs also transduce signals for three basic taste qualities of sweet, umami (savory taste), and bitter, as well as the flavor sensation kokumi. Taste GPCRs reside in specialised taste receptor cells (TRCs) within taste buds. Type I taste GPCRs (TAS1R) form heterodimeric complexes that function as sweet (TAS1R2/TAS1R3) or umami (TAS1R1/TAS1R3) taste receptors, whereas Type II are monomeric bitter taste receptors or kokumi/calcium-sensing receptors. Sweet, umami and kokumi receptors share structural similarities in containing multiple agonist binding sites with pronounced selectivity while most bitter receptors contain a single binding site that is broadly tuned to a diverse array of bitter ligands in a non-selective manner. Tastant binding to the receptor activates downstream secondary messenger pathways leading to depolarization and increased intracellular calcium in TRCs, that in turn innervate the gustatory cortex in the brain. Despite recent advances in our understanding of the relationship between agonist binding and the conformational changes required for receptor activation, several major challenges and questions remain in taste GPCR biology that are discussed in the present review. In recent years, intensive integrative approaches combining heterologous expression, mutagenesis and homology modeling have together provided insight regarding agonist binding site locations and molecular mechanisms of orthosteric and allosteric modulation. In addition, studies based on transgenic mice, utilizing either global or conditional knock out strategies have provided insights to taste receptor signal transduction mechanisms and their roles in physiology. However, the need for more functional studies in a physiological context is apparent and would be enhanced by a crystallized structure of taste receptors for a more complete picture of their pharmacological mechanisms.

## Introduction

G protein-coupled receptors (GPCRs) are the largest and the most diverse group of membrane receptors in eukaryotes. They are activated by a wide variety of ligands in the form of light energy, lipids, sugars, peptides and proteins (Billington and Penn, 2003; Schoneberg et al., 2004; Lundstrom, 2009) which convey information from the outside environment into the cell to mediate their corresponding functional responses. The conformational changes of GPCRs upon ligand binding initiate a series of biochemical reactions within the cell. These intracellular reactions regulate sensory functions of smell, taste, and vision, and a wide variety of physiological processes such as secretion, neurotransmission, metabolism, cellular differentiation, inflammation and immune responses (Lagerström and Schiöth, 2008; Rosenbaum et al., 2009; Venkatakrishnan et al., 2013; Ahmad et al., 2015). Taste is one of the most important sensations for human life, enabling us to perceive different tastes from the diverse range of food available in nature and is a major determinant of our ingestion decisions.

**FIGURE 1:**
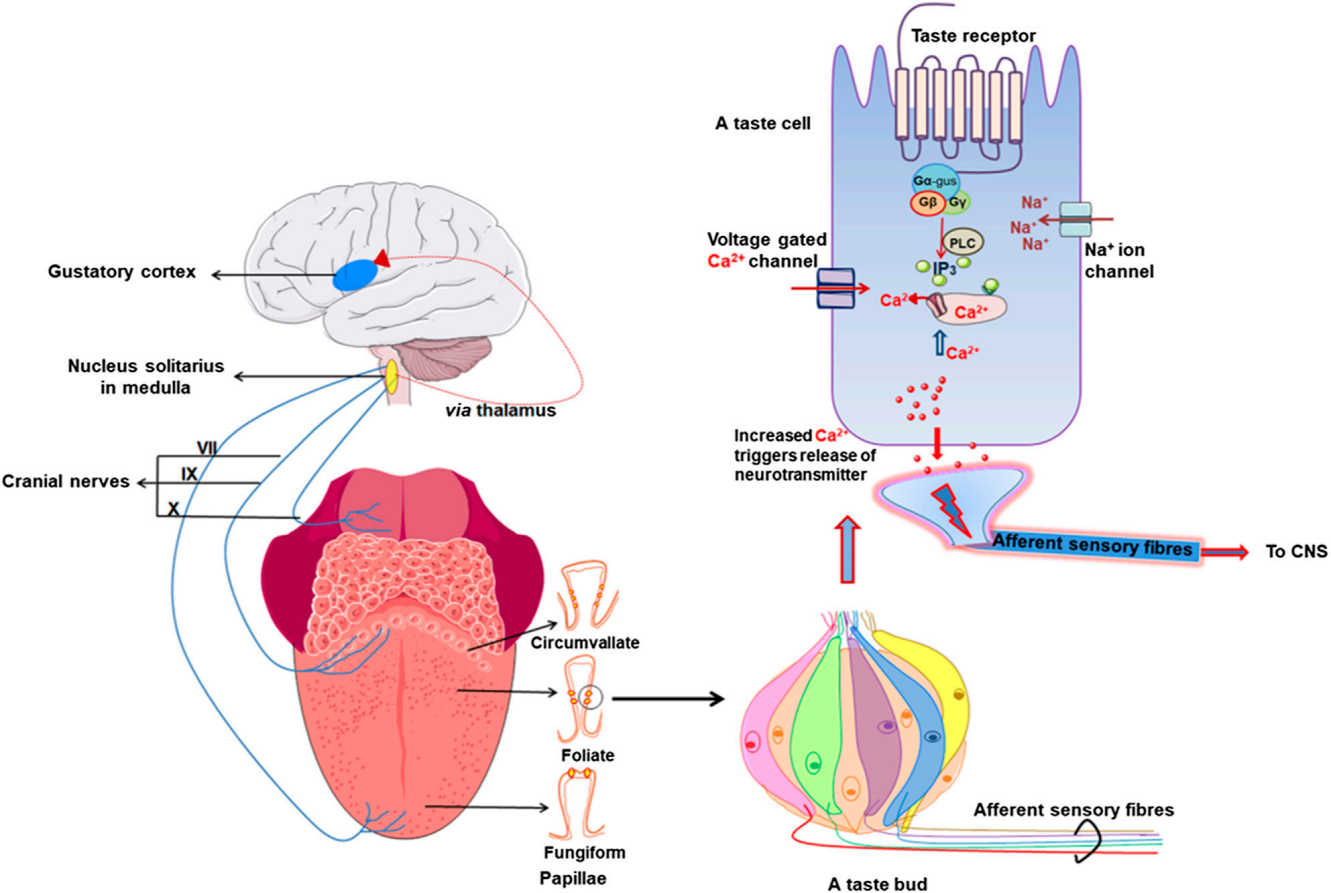
Schematic diagram shows taste signal transmission between tongue and brain. Taste buds present in different papillae in tongue and palate contain taste receptor cells (TRC) which contain taste G protein-coupled receptors (GPCRs). Left side shows how afferent nerves transmit a signal to the gustatory cortex in brain via cranial/glossopharyngeal nerves. Right side shows taste bud with taste TRCs and simplified signal transduction pathway of taste receptor where taste GPCRs are activated by a tastant that in turn recruits a specific G protein that further induces intracellular calcium release (created with BioRender.com).

The anatomical units of taste detection are taste receptor cells (TRCs) that are assembled into taste buds distributed across different papillae of the tongue and palate epithelium. Taste processing is first achieved at the level of TRCs that are activated by specific tastants. They transmit information *via* sensory afferent fibers to the gustatory cortex in the brain for taste perception (Figure 1). Three different morphologic subtypes of TRCs in taste buds sense the different tastes we perceive. Type I glial-like cells detect salty taste while type II cells expressing GPCRs detect sweet, umami, and bitter tastes. Type III cells sense sour stimuli (Janssen and Depoortere, 2013).

**FIGURE 2:**
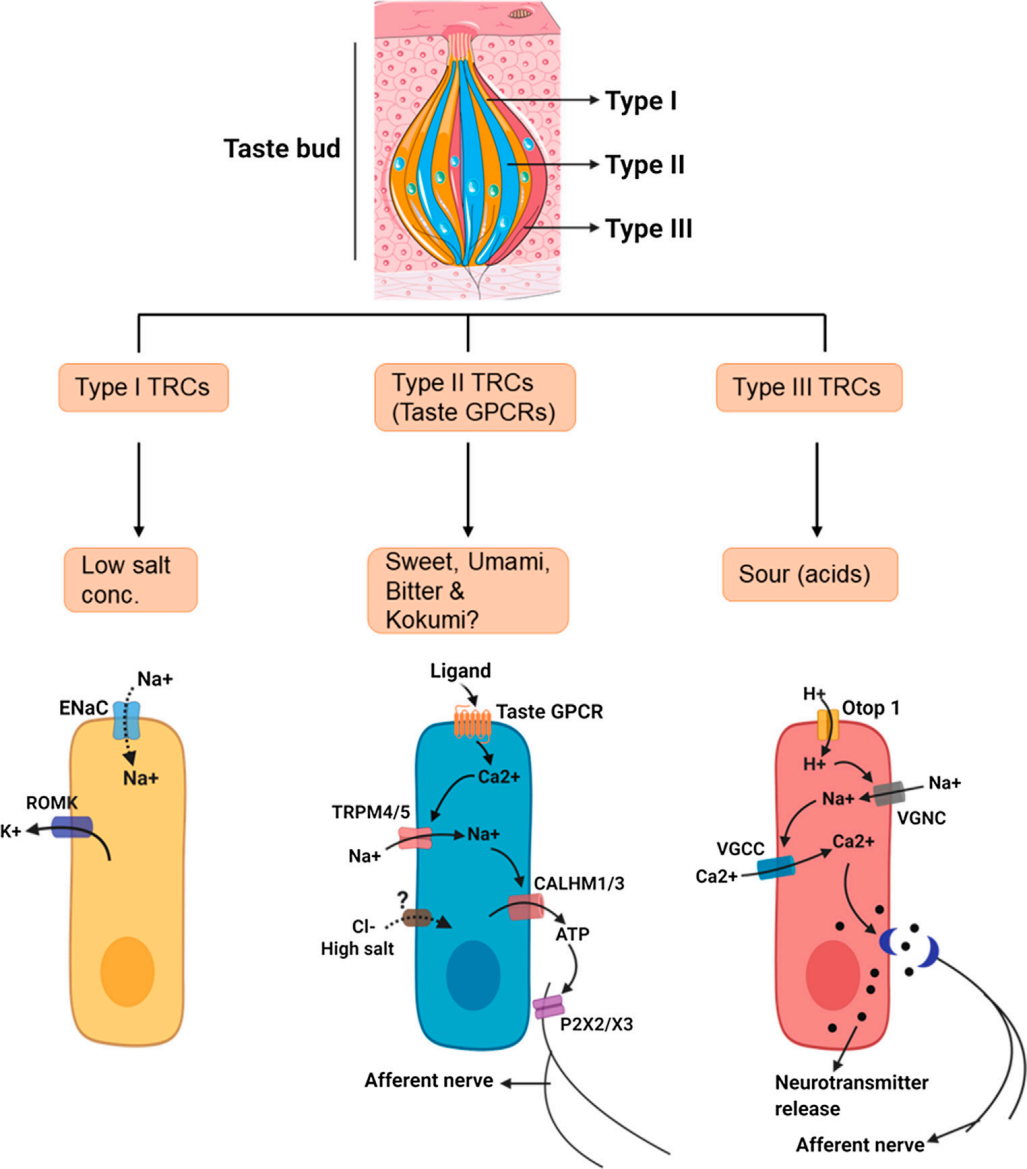
Schematic representation of different types of taste receptor cells (TRCs) in taste bud with their attributed taste modalities and signal transduction. Type I TRCs exhibit a support function similar to glial cells and express enzymes and transporters that remove extracellular neurotransmitters (Lawton et al., 2000; Bartel et al., 2006; Vandenbeuch et al., 2013), and ion channels linked with the redistribution and spatial buffering of K+ (Dvoryanchikov et al., 2009). A subpopulation of type I cells are thought to be involved in low salt taste perception (Vandenbeuch et al., 2008) but this remains to be confirmed. Type II TRCs are receptor cells and express G protein-coupled receptors (GPCRs) on their surface that respond to sweet, umami and bitter tasting stimuli. The type II TRCs are fine-tuned and express either type 1 (TAS1R2/TAS1R3: sweet and TAS1R1/TAS1R3: umami) or type 2 taste (TAS2Rs; bitter) GPCRs and correspondingly respond to sweet/umami or bitter stimuli (Matsunami et al., 2000; DeFazio et al., 2006; Yoshida et al., 2009) (see also Table 2 for classification). Moreover, three isoforms of type 1 taste GPCRs (TAS1R1, TAS1R2 and TAS1R3) are often co-expressed and responses to both sweet and umami stimuli can be detected in the same cell (Kusuhara et al., 2013). Interestingly, recent studies reported a novel subpopulation of cells with type II TRCs that transduce a signal in response to high salt concentrations (>150 mM) (AI) (Roebber et al., 2019). Type III TRCs are the least abundant and sense sour stimuli through the proton selective channel, otopterin 1 (Tu et al., 2018; Zhang et al., 2019). As a consequence of expressing several synaptic proteins, they are termed presynaptic cells (DeFazio et al.,2006). Although both Type II and Type III TRCs require action potentials for transmitter release, their working mechanisms are quite different. Whereas, type III TRCs use a conventional synapse and SNARE mechanism like that in neurons to affect the release of synaptic vesicles, type II TRCs rely on action potentials to trigger the release of ATP through voltage gated channels (DeFazio et al., 2006; Vandenbeuch et al., 2013) (see also Figures 1, 3) (created with BioRender.com).

**FIGURE 3:**
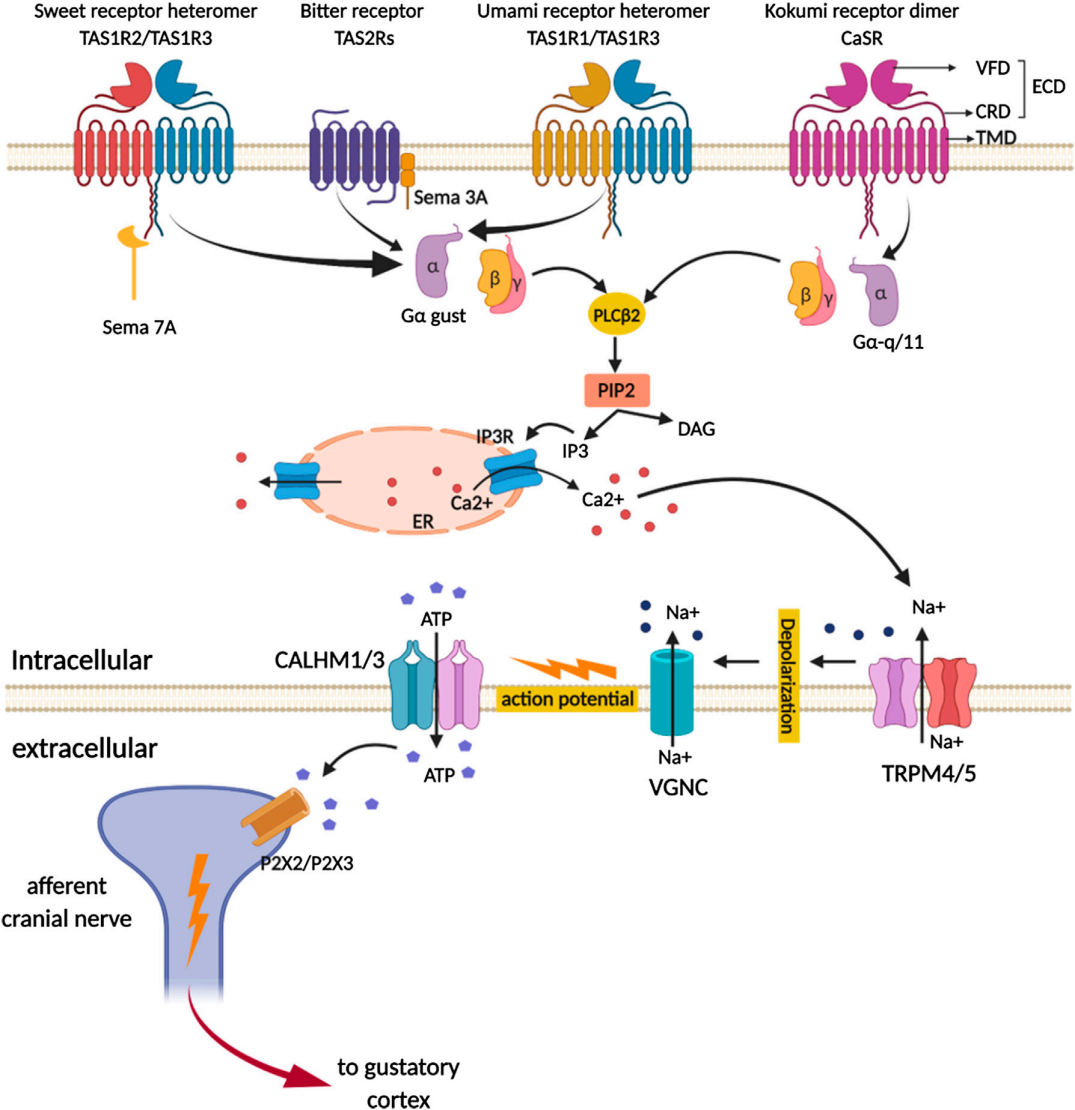
Schematic representation of signal transduction pathway of sweet, umami, bitter and kokumi-calcium sensing receptors (CaSR) in taste receptor cells on the tongue. Ligand-induced stimulation of the sweet (TAS1R2/TAS1R3), umami (TAS1R1/TAS1R3), bitter receptors (TAS2Rs) and kokumi sensation expressed in type II taste cells within taste bud activates a trimeric G protein composed of α-gustducin (Gα-gust) in sweet, umami, bitter and Gα-q/11 in kokumi-receptor and a complex consisting of Gβγ proteins. The released Gβγ-complex activates phospholipase C isoform β2 (PLCβ2) which then induces production of inositol 1,4,5-trisphosphate (IP3) and diacylglycerol (DAG); the second messenger IP3, in turn activates the IP3 receptor (IP3R), an intracellular ion channel that allows Ca^2+^ release from the intracellular endoplasmic reticulum (ER store). Increase in intracellular Ca^2+^ then activates the complex of transient receptor potential cation channel subfamily M member 4 and 5 (TRPM4/5) that are plasma membrane localized sodium-selective channels which leads to depolarization and subsequent activation of voltage-gated sodium channels (VGSC). The combined action of increased Ca^2+^ and membrane depolarization activates the complex of calcium homeostasis modulator 1 and 3(CALHM1/3) channel and pannexin1 channels, thus resulting in the release of the neurotransmitter ATP. Increased ATP, in turn activates P2X ionotropic purinergic receptors 2 and 3 (P2X2/P2X3) on afferent cranial nerve generating an action potential that subsequently signals to the gustatory cortex for sensory perception. Besides well-known taste GPCR pathways, connecting proteins semaphorin 7A (Sem 7A) and 3A (Sem 3A) are depicted in close contact with sweet and bitter receptors as they provide instructive signals that fine tune to sweet or bitter ganglion neurons, respectively. VFT, venus flytrap domain; CRD, cystine rich domain; ECD, extracellular domain. (created with BioRender.com).

Sweet and umami stimuli are transduced by Type 1 taste GPCRs while bitter taste is sensed by Type 2 taste GPCRs (Figure 2; Table 1). The more recently described kokumi sensation is mediated by another GPCR, the calcium-sensing receptor (CaSR) (Figure 2; Table 1). Taste GPCRs are activated by specific taste ligands present in foods and recruit G proteins to activate downstream signaling effectors (Figure 3).

**TABLE 1:** Taste GPCRs classification and their downstream signaling regulators.

Taste/flavor sensation	Sweet	Umami	Bitter	Kokumi
GPCRs	TAS1R2 + TAS1R3	TAS1R1 + TAS1R3	TAS2Rs	Calcium sensing receptor
Class of GPCR	Class C	Class C	Class A	Class C
Type	Type 1 taste GPCRs	Type 1 taste GPCRs	Type 2 taste GPCRs	ND
Oligomerization	Heterodimer	Heterodimer	Monomer	Homodimer
Gα Subunit	Gα-gustducin	Gα-gustducin	Gα-gustducin	Gq/11, Gi/o, G12/13, Gs
Signaling pathway	Gβγ-PLCβ2-TRPM5 pathway- semaphorin 7A	Gβγ-PLCβ2-TRPM5 pathway	Gβγ-PLCβ2-TRPM5 pathway-semaphorin 3A	Gβγ-PLCβ2-TRPM5 pathway
Ligand binding sites	VFT; TMD	VFT, TMD	ND	VFT, TMD
Potent ligands	Sucrose, aspartame, sucralose, cyclamate	L-amino acids, Gultamate	Peptides, alkaloids, flavonoids	Ca^2+^, divalent and trivalent cations, Glutathione, γ-glutamyl peptides, polyamines, amino acids
Antagonists/NAMs	Lactisole, gymnemic acid	Lactisole, clofibric acid	GIV3727, probenecid	NPS2143
Food source	Sugar/sweets, dairy products	Savoury food, fermented dairy/meat/fish/chicken broth, mushroom, tomato	Wine, tea, coffee, cheese, broccoli	Garlic/onion meat/fish/broth/mushroom, soy sauce

TAS2R, bitter taste receptors; GPCR, G protein-coupled receptors; VFT, extracellular Venus flytrap domain; TMD, seven transmembrane domain; ND, not defined.

In this review, we will first explore the basic architecture of the gustatory sensory system and its peripheral signal transmission. Then we will discuss taste GPCR signal transduction mechanisms for the different taste modalities, their molecular structure, and the conformational changes that occur following orthosteric/allosteric binding of endogenous and food-derived ligands.

## Taste Buds and Neural Transmission

In mammals, taste buds on the tongue comprise 50–100 elongated epithelial cells and a small number of proliferative basal cells (Sullivan et al., 2010). Ultrastructural studies and patterns of gene expression with cell function reveal three distinct anatomical types of TRCs within each taste bud: Type I, Type II and Type III (Murray, 1986) (refer to Figure 2; Table 2).

**TABLE 2:** Summary of taste receptor cell characteristics.

Taste receptor cells	Type I	Type II	Type III
Cell type	50% of the total population	20–30%	5–10%
Taste responses	Low salt taste amiloride sensitive response	Sweet, biiter, umami and probably kokumi flavor response (?) via GPCRs	Sour response via otopterin 1
Morphology	Spindle shaped with long brush like microvilli (1–2 mm), no synapse	Long and slender short microvilli, no synapse	Single large microvillus, have synaptic contact with afferent nerve
Other functions	Support function, ion redistribution, neurotransmitter clearance	High salt taste amiloride insensitive response?	Probable salt response?
Marker proteins	GLAST, K^+^ channel (ROMK)	CALHM1, CALHM3, pannexin, connexins	Kir2.1, PDK2L1, SNAP25, synapsinII, NCAM

GLAST, glutamate aspartate transporter; ROMK, renal outer medullary potassium channel; PDK2L1, polycystin 2 like 1, transient receptor potential cation channel; CALHM, calcium homeostasis modulator; Kir2.1, inward rectifier K(+) channel; NCAM, neural cell adhesion molecule; SNAP25, synaptosome associated protein 25 kDa.

Type II TRCs express either sweet, umami, or bitter taste receptors at their cell surface. These receptors share some commonality to their signal transduction mechanisms that are intrinsic to TRCs. Taste GPCRs (sweet, umami and bitter) couple to heterotrimeric G proteins that include Gα-gustducin, Gβ3, and Gγ13 (Huang et al., 1999) and initiate a series of signal transduction cascades involving activation of phospholipase C-β2 (PLCB2), production of inositol-1,4,5-triophosphate (IP3), and IP3-dependent Ca^2+^ release from the endoplasmic reticulum (ER) via the IP3 receptor (IP3R). The increased intracellular [Ca^2+^]_i_ then activates the transient receptor potential cation channel subfamily M member 4 and 5 (TRPM4/5) in the basolateral plasma membrane, leading to membrane depolarization that triggers Na^+^ action potential firing, and depolarization-induced release of ATP. In turn, ATP acts as the primary neurotransmitter stimulating purinergic receptors 2 and 3 (P2X2 and P2X3) on afferent cranial nerves whose activation triggers an action potential which subsequently activates the gustatory cortex in the brain (McLaughlin et al., 1992; Wong et al., 1996; Margolskee, 2002). α-gustducin is a distinct G protein selectively expressed in ∼30% of type II TRCs and shares 80% identity with retinal protein α-transducin (McLaughlin et al., 1992) and is a key contributor to signal transduction for sweet and bitter taste receptors (McLaughlin et al., 1992; Wong et al., 1996; Margolskee, 2002).

An important aspect of taste transduction is how ATP signaling is conducted. Recent studies have discovered that calcium homeostasis modulators 1 and 3 (CALHM1/3) are enriched in type II TRCs where they interact and form a functional complex. Their genetic deletion abolishes responses to sweet, bitter and umami tastes, supporting the requirement of the CALHM1/3 complex as an ATP release channel for the GPCRs mediated tastes (Taruno et al., 2013; Ma et al., 2018).

New information has provided insight into how specific taste qualities are fine-tuned in order to recognize their partner ganglionic neurons in the brain. Lee et al. (2017) discovered semaphorin proteins, 7A and 3A as the physical links between sweet and bitter TRCs, respectively, and their partner ganglion neurons in the brain. It remains to be determined what physical links exist between umami TRCs and their corresponding neurons in the brain. Delineating the underlying molecular basis for this interaction would provide further understanding of purinergic transmission in the taste system. In addition, whether these mechanisms are relevant for kokumi sensation has not yet been investigated, despite CaSR having distinct expression in TRCs and significant functional synergy with other prominent taste qualities (sweet, umami and salty). Moreover, there is still debate regarding the recognition of kokumi as a sixth taste entity, consequently the calcium sensing receptor (CaSR) is not yet included in the nomenclature for any subtypes of taste GPCRs, although it would best fit with Type 1 taste receptors.

### Type 1 Taste G Protein-Coupled Receptors (Sweet and Umami)

The type 1 taste receptors (TAS1Rs) belong to the class C GPCRs, which possess a large N-terminal extracellular domain (ECD) fused to the heptahelical seven transmembrane domain (TMD). The ECD is further divided into two ligand-binding domains (LBD1 and LBD2), having a bi-lobed structure called a Venus flytrap domain (VFT) due to its resemblance to this shape (Hoon et al., 1999). With the exception of GABA_B_ receptors, a cysteine-rich domain (CRD) connects the VFT to the TMD (Leach and Gregory, 2017).

**FIGURE 4:**
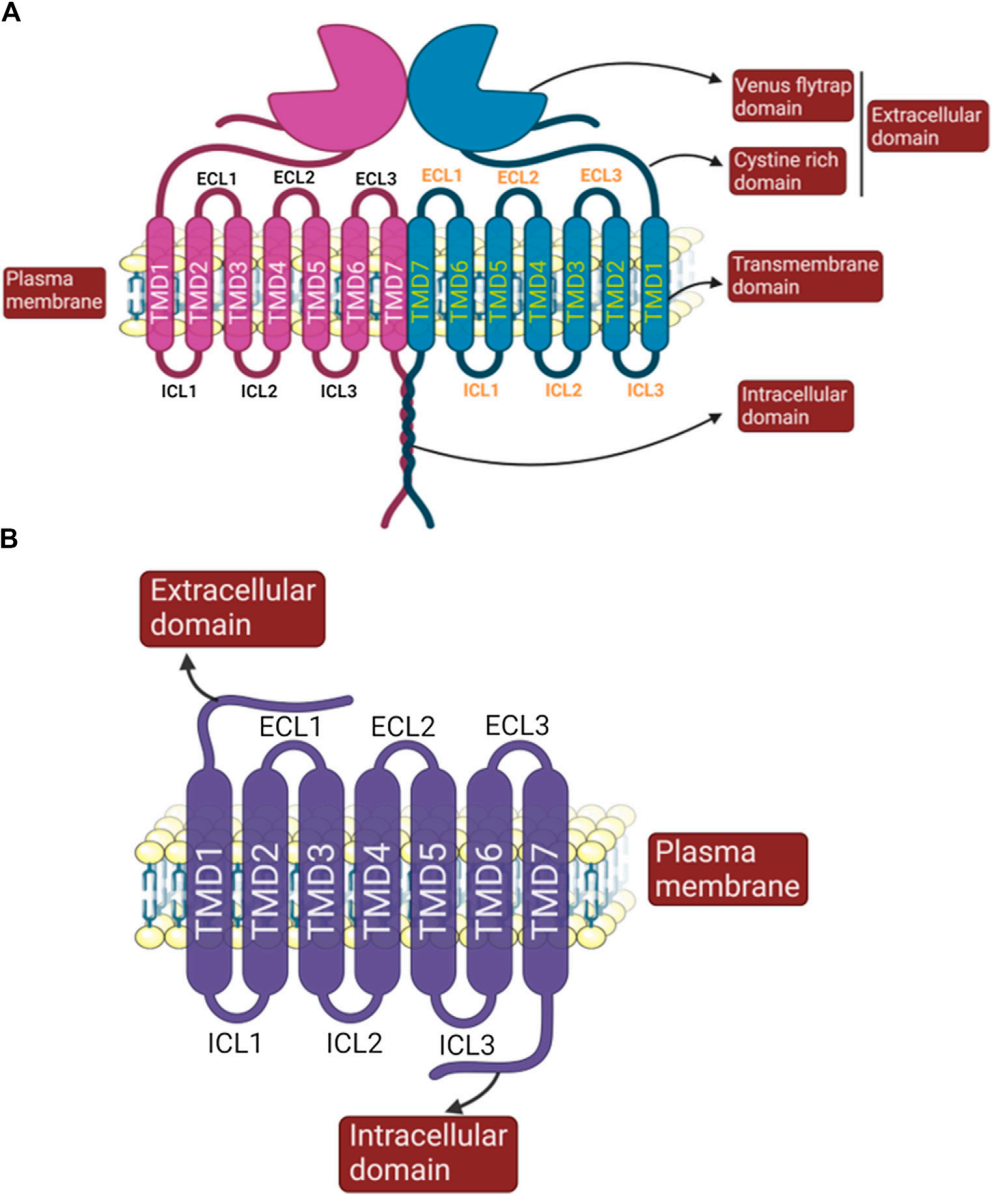
Figure depicting basic structural features of sweet/umami/kokumi dimeric receptor **(A)** and monomeric bitter receptor **(B)** (created with BioRender.com).

In contrast to other receptors from this class C of GPCRs, such as the metabotropic glutamate receptor (mGluR) or γ-aminobutyric acid type B receptors (GABABRs) which function as homo- or heterodimers, respectively (Jones et al., 1998; Kaupmann et al., 1998; White et al., 1998; Kunishima et al., 2000), the TAS1Rs function as obligatory heterodimers. The distinct expression pattern of TAS1R1 and TAS1R2 in different subsets of murine cells led to the idea that they could detect two different taste profiles. However, following the discovery of the TAS1R3 subtype, it was clear that when TAS1R1 heterodimerizes with TAS1R2, the receptor detects sweet taste substances (Nelson et al., 2001; Ohkuri et al., 2009; Kim et al., 2017). On the other hand, if heterodimerized with TAS1R3 (TAS1R1/TAS1R3), it is responsible for umami or amino acid taste detection (Li et al., 2002; Nelson et al., 2002). Please refer to figure 4A for the basic structure of sweet and umami receptors.

## Sweet Taste Signal Transduction Mechanisms

The TAS1R2/TAS1R3 receptor recognizes a wide variety of sweet substances including natural sugars, artificial sweeteners, amino acids and proteins (Li et al., 2002; Xu et al., 2004; Jiang et al., 2005a; Jiang et al., 2005c) (Table 3). This was demonstrated in studies using heterologous expression systems as well as knockout mice for TAS1R2 and/or TAS1R3 subtypes that showed a blunted response to sugars, sweeteners, and D-amino acids, confirming the TAS1R2/TAS1R3 heterodimer as the main sweet taste receptor *in vivo* (Li et al., 2002; Zhao et al., 2003; Xu et al., 2004).

**TABLE 3:** Agonists of sweet taste receptor along with their EC50 values.

Agonists	Nature	Binding pocket	EC50 (mM)	References
Sucrose	Natural carbohydrate	VFT (TAS1R2 and TAS1R3)	62	(Li et al., 2002; Servant et al., 2010; Zhang et al., 2010; Zhang et al., 2003)
Aspertame	Peptide	VFT (TAS1R2)	0.75	(Li et al., 2002; Liu et al., 2011; Masuda et al., 2012)
Neotame	Peptide	VFT (TAS1R2)	5	(Li et al., 2002; Masuda et al., 2012)
Cyclamate	Sulfamate	TMD (TAS1R3)	3.1	(Xu et al., 2004; Jiang et al., 2005c)
Brazzein	Protein	CRD (TAS1R3)	0.08	(Li et al., 2002; Jiang et al., 2004; Ide, et al., 2009; Masuda et al., 2012)
Thaumatin	Protein	CRD (TAS1R3)	0.005	Masuda et al., 2012; Jiang et al., 2004
Monellin	Protein	VFT (TAS1R3), VFT (TAS1R2)	0.01	Koizumi et al., 2007; Jiang et al., 2004
Neoculin	Protein	VFT (TAS1R2)	0.001	(Jiang et al., 2004; Koizumi et al., 2007)
Saccharin	N sulfonyl amide	VFT (TAS1R2)	0.19	(Li et al., 2002; Masuda et al., 2012; DuBois, 2016)
Suosan, cyanosuasan	Arylurea	VFT (TAS1R2)	ND	(Tinti and Nofre, 1991; Du Bois, 2016)
SC-45647	Guanidinoacetic acid	VFT (TAS1R2)	0.3	(DuBois, 1995; Sanematsu et al., 2014)
Sucralose	Halogenated carbohydrate	VFT (TAS1R2 and TAS1R3)	0.06	(Li et al., 2002; Masuda et al., 2012)
Acesulfame K	Sulfamate ester	VFT (TAS1R2)	0.54	(Li et al., 2002; Masuda et al., 2012)
Perillartine	Oxime, ethoxyphenyl urea, alkoxyaryl urea,	TMD (TAS1R2)	15	(Li et al., 2002; Servant et al., 2010)
Dulcin	Ethoxyphenyl urea	TMD (TAS1R2)	0.01	(Servant et al., 2010)
S819	Alkoxyaryl urea	TMD (TAS1R2)	0.025	(Zhang et al., 2008)
D-tryptophan	Amino acid	VFT (TAS1R2)	2.09	(Li et al., 2002; Masuda et al., 2012)
Xyletol, sorbitol	Polyols	VFT (TAS1R2)	ND	(Mahalapbutr et al., 2019)
Maltotriose, acarbose	Oligosaccharide, pseudotetrasaccharide	ND	ND	(Pullicin et al., 2017; Pullicin et al., 2019)

Where VFT, venus flytrap domain; TMD, transmembrane domain; ND, not determined.

The sweet receptor couples to heterotrimeric Gα-gustducin which includes Gβ3 and Gγ13, as mice lacking Gα-gustducin showed a reduced response to sweet substances either natural or artificial (McLaughlin et al., 1992; Wong et al., 1996; Margolskee, 2002). Moreover, a point mutation in the C-terminal region of gustducin (G352P) (critical for its receptor interaction) results in loss of its ability to activate taste GPCRs while keeping other functions intact. Further, G352P acts as a dominant negative to block heterotrimeric G protein interaction with taste receptors and disrupts the responses to sweet and bitter compounds in both wild type (WT) and null mice (Ruiz-Avila et al., 2001). In addition, the G352 mutant further reduces any residual sweet/bitter taste responses of the null mice by acting as a “βγ sink” to bind all unbound βγ-subunits and remove them from the viable pool of G protein heterotrimers available to the receptor (Ruiz-Avila et al., 2001). These observations confirm the essential requirement of Gα-gustducin in sweet and bitter taste transduction.

In addition to the Gα-gustducin pathway, sweet taste transduction occurs *via* two additional signaling pathways involving different secondary messengers. The first one involves cAMP and the second one involves IP3. Normally, sugars elevate the level of cAMP, while sweeteners stimulate IP3 production (Tonosaki and Funakoshi, 1988; Uchida and Sato, 1997). Sucrose or other sugars bind to either TAS1R2 or TAS1R3 and recruit Gαs protein that leads to increased cAMP levels which initiates the influx of cations through ion channels. Alternatively, cAMP activates protein kinase A that leads to TRC cell depolarization resulting in an influx of calcium ions and neurotransmitter release (Avenet et al., 1988; Tonosaki and Funakoshi, 1988; Margolskee, 2002). Sweetener binding to the TAS1R2/TAS1R3 heterodimer recruits Gα-gustducin proteins that stimulate PLCβ2 which in turn hydrolyzes phosphatidylinositol 4,5-bisphosphate (PIP2) to diacylglycerol (DAG) and IP3 (Margolskee, 2002; Chandrashekar et al., 2006). IP3R3 (Hisatsune et al., 2007) induced Ca^2+^ release from ER stores (Figure 3) activates TRPM5 (Zhao et al., 2003; Hisatsune et al., 2007; Dutta Banik et al., 2018) that leads to an action potential (Yoshida et al., 2005; Yoshida et al., 2006) and subsequent release of neurotransmitters.

Interestingly, Dutta Banik et al. (2018) confirmed that TRPM4 also mediates taste signaling independent of TRPM5, and knocking out both channel proteins (TRPM4/5) abolishes the sweet, umami and bitter taste response completely. This revealed another layer of complexity to sweet signal transmission. This in-depth mechanistic research has increased our understanding of sweet and bitter receptors and presents a challenge to dissect the taste signal transmission pathways for umami and kokumi as well.

### Structural, Molecular and Conformational Changes of Sweet Receptor

Since, the sweet taste receptor has not yet been crystallized, determining the structure of the sweetener binding site and mechanism of activation has been a challenge. Based on homology with other class C GPCRs (mGluRs and GABA_B_Rs), multiple studies propose similar activation mechanisms for the sweet receptor (Kunishima et al., 2000; Tsuchiya et al., 2002; Jingami et al., 2003; Muto et al., 2007; Perez-Aguilar et al., 2019). The many different sweet agonists and their diverse binding sites across receptor domains (VFT, TMD and CRD) (Table 3) may explain its complex yet broadly tuned nature. For example, a single residue in VFT (I60) of TAS1R3 of the TAS1R2/TAS1R3 heteromer is required for a saccharin preference in in-bred mouse strains (Max et al., 2001; Reed et al., 2004).

Several studies utilizing homology and computational modeling based on the crystal structure of mGluR and GABA_B_Rs have predicted structural and functional aspects of orthosteric and allosteric binding sites for the sweet receptor (Kim et al., 2017; Cheron et al., 2019; Park et al., 2019). They reported that both VFT regions undergo ligand dependent conformational changes and intersubunit interactions between ECDs that further stabilize heterodimer formation for subsequent downstream signaling (Perez-Aguilar et al., 2019). Binding of orthosteric agonists to VFT of TAS1R2 leads to major conformational changes that form a TMD6/TMD6 interface between TMDs of TAS1R2 and TAS1R3, which is consistent with the activation process observed biophysically on the mGluR2 homodimer. The initial role of the bound agonist is to pull the bottom part of VFT3 (VFT of TAS1R3) toward the bottom part of VFT2 (VFT of TAS1R2) in order to transmit this movement from VFT2 (where agonists bind) through the VFT3 and the CRD3 (VFT and CRD of TAS1R3) to the TMD3 (TMD of TAS1R3). This facilitates G protein coupling and downstream signaling. The CRDs are crucial in this streamlined relay of structural changes where disulfide bonds provide rigidity to the CRD and amplify the mechanical constraints that help in attaining an active conformation (Cheron et al., 2019). This is empirically supported by a study in which a single mutation (A537P) in the CRD of TAS1R3 abolished the response to all sweeteners, indicating that the CRD3 must couple ligand binding in VFT2 to the conformational changes required in TMD3 for receptor activation.

Trafficking and cell surface expression are also crucial factors for sweet taste transduction. Molecular modeling with mutagenesis scanning revealed specific regions consisting of hydrophobic residues in ECD (site II; at the tip of CRD) and TMD regions (site IV; includes TMD6 and the cytoplasmic base of TMD5) of the TAS1R2 subunit to be important for dimerization with TAS1R3. Moreover, the CRD region and ECL2 domain of the transmembrane region seem to be important for surface co-expression of the TAS1R2/TAS1R3 dimer. In particular, the cytosolic C-terminus portion of the CRD region of TAS1R2 needs to be properly folded for coexpression and trafficking (Park et al., 2019). This reflects the difficulty in expressing these receptors at consistent levels in mammalian cell lines (Li et al., 2002; Shimizu et al., 2014).

#### Positive Allosteric Modulation of Sweet Receptor

Class C GPCRs pose an ideal target for allosteric modulation either positive (PAM) or negative (NAM). PAMs show little or no agonist activity on their own but significantly enhance agonist activity. Sweet taste is a major target of the food industry globally and non-caloric sweeteners are highly sought to exploit a huge commercial market. In a first comprehensive high throughput screen by Servant et al. (2010), novel PAMs (SE1, SE2, SE3; Table 4) for the sweet heteromer were reported that were not sweet on their own but significantly enhanced the sweetness of sucralose or sucrose. Agonist binding to the VFT region of TAS1R2, facilitates a closed conformation which constitutes an active state of the sweet receptor, while its open conformation represents an inactive state. Molecular modeling and mutagenesis studies revealed that these PAMs follow a similar mode of binding as that reported for umami PAMs (IMP and GMP). They bind near the opening of the binding pocket of the VFT region adjacent to their agonists, through Van der Waals and hydrogen bonding interactions, and utilize several critical residues for their activity. Although these residues are not in direct contact with any receptor bound sweetener, mutation of some of them (K65, Y103, L279, D307, and R383) diminishes the response to sweeteners suggesting that these residues normally stabilize the closed conformation. Initial closing of the VFT region by agonist binding and further stabilization of the closed conformation by subsequent binding of SE modulators occurs in two steps. First, by interacting with the ECD region of TAS1R2, and second, by strengthening the hydrophobic interactions between the two lobes of ECD and lowering the free energy needed for their closure (Zhang et al., 2010).

**TABLE 4:** Sweet taste receptor’s positive allosteric regulators with concentration (used in cell based assays in studies) and negative allosteric modulators with their IC50 values.

Positive allosteric modulators (PAMs)	Nature	Binding pocket	Conc. (mM)	References
SE1, SE2, SE3	Undisclosed	VFT (TAS1R2)	0.05	(Servant et al., 2010; Zhang et al., 2010)
Neohesperidin dihydrochalcone (NHDC)	Flavonoid	TMD (TAS1R3)	0.25	(Jiang et al., 2005c; Winnig et al., 2007)
Unnatural tripeptides (several)	Biaryl derivative tripeptides	ND	2 – 20	Yamada et al., 2019
Sodium, cholestrol	Cation, lipid	TMD (TAS1R2)	ND	Perez-Aguilar et al., 2019
**NAMs**	—	—	**IC50 (mM)**	—
Lactisole	Carboxylic acid salt	TMD (TAS1R3)	0.041	(Jiang et al., 2005c)
(2-(2,4-dichlorophenoxy)propionic acid)	Carboxylic acid salt	TMD (TAS1R3)	0.006	(Nakagita et al., 2019)
Gymnemic acid	Triterpenoid glycoside	TMD (TAS1R3)	6.9	(Sanematsu et al., 2014)
Clofibric acid	Herbicide	TMD (TAS1R3)	1.4	(Maillet et al., 2009; Kochem and Breslin, 2017)
Amiloride	Diuretic	TMD (TAS1R2)	0.87	(Imada et al., 2010; Zhao et al., 2018)
Umami compounds: MSG, Glu-Glu, Glu-Asp	Peptides	VFT (TAS1R2)	ND	(Shim et al., 2015)

Where VFT, Venus flytrap domain; TMD, transmembrane domain; ND, not determined.

Using a high throughput chemical screening approach and heterologous expression of the TAS1R2/TAS1R3 heteromer, several unnatural tripeptides with a novel core biaryl structure were found as potential sweet enhancers (Yamada et al., 2019). This study divided the potential molecule into three parts namely, “head and linker” which together are essential for its sweet enhancer activity, while the “tail” determines the level of activity. This approach provided some useful inputs toward synthesis of potent PAMs. Firstly, an amine incorporated at the α-position of carbonyl moiety in the tail structure interacts with the TAS1R2 subunit thereby increasing allosteric activity. Secondly, additional hydrophobic substitutions in the tail structure provided increased allosteric activity to the molecule. Lastly, distance between the head and linker and insertion of an amide bond is crucial for its synthesis. Although, their binding characteristics and allosteric mechanisms are not yet known, these observations provide a starting point to identify and synthesize new sweet PAMs in the future.

Small molecule PAMs can also bind to the transmembrane domain in class C GPCRs, in contrast to agonist which binds to the extracellular domain (Urwyler, 2011). For example, the flavonoid sweetener, neohesperidin dihydrochalcone (NHDC) binds to TMD regions to enhance the agonist induced sweet response. It interacts with a receptor binding pocket in the TMD of TAS1R3 and requires seventeen critical residues in TMDs and extracellular loop 2 for its allosteric activity (Winnig et al., 2007). These residues also contribute to cyclamate and lactisole binding sites. Among seventeen residues, eight alter receptor activation by NHDC (Q637^3.29^, S640^3.32^, H641^3.33^, Y699^4.60^, W775^6.48^, F778^6.51^, L782^6.55^, and C801^7.39^) and influence lactisole mediated inhibition. Similarly, nine of the seventeen residues (Q637^3.29^, H641^3.33^, H721^ex2^, S726^5.39^, F730^5.43^, W775^6.48^, F778^6.51^, L782^6.55^, and C801^7.39^) mediate activation by cyclamate, while six (Q637^3.29^, H641^3.33^, W775^6.48^, F778^6.51^, L782^6.55^, and C801^7.39^) influence receptor inhibition by lactisole as well as receptor activation by cyclamate [superscript refers to the nomenclature suggested for class C GPCRs by Pin et al. (2003) where first number denotes TMD region and the second number denotes residue position from the most conserved residue].

Notably, three critical residues in TMD6 (W775^6.48^, F778^6.51^, L782^6.55^) and one in TMD7 (C801^7.39^) of TAS1R3 were found crucial for allosteric binding, as their mutation to alanine altered the receptor's sensitivity to NHDC and cyclamate, as well as to the inhibitor lactisole (Winnig et al., 2005). Therefore, TMD6 and TMD7α helices of TAS1R3 are integral to allosteric modulation of the sweet receptor, implicating them in TAS1R2 and TAS1R3 subunit interactions and indicating an important role for this structural region in the conformational changes involved in receptor activation. Furthermore, these residues are conserved across mammalian species (Cheron et al., 2019).

#### Negative Allosteric Modulation of Sweet Receptor

Like PAMs, negative allosteric modulators (NAM) such as lactisole and gymnemic acid bind to the TMD region of TAS1R3 and inhibit sweet substance induced responses. Lactisole, an aralkyl carboxylic acid not only inhibits sweet but also the umami receptor response in humans and presents a rare opportunity to study the structural cross talk between these two taste qualities. Using heterologous expression and mutagenesis, Jiang et al. (2005b) reported that lactisole's sweet inhibition might be mediated by its binding to TMD3, TMD5, and TMD6 of TAS1R3 and induce a conformation change which restricts the movement required to stabilize the active state. Residues A733^5.46^ in TMD5, L798^7.36^ in TMD7, and R790^ex3^ in extracellular loop 3 were found to be crucially important for sensitivity to lactisole in humans (Jiang et al., 2005b). These observations were confirmed in a recent study where 2-(2,4-dichlorophenoxy)propionic acid (2,4-DP) was found to be a more potent antagonist and utilise the same residues as well as four additional ones (H641^3.37^, H734^5.43^, F778^6.53^ and Q794^7.32^) in binding to TAS1R3. Moreover, the (S)- isomer of both compounds was found to be more strongly bound to the TMD of TAS1R3 and be a more effective inhibitor [lactisole; (S)-lactisole IC50, 20 µM while (R)- lactisole exerted no inhibition at this conc.; 2,4-DP: (S)-isomer was 10 fold more effective than (R)-2,4DP]. The (S)- lactisole isomer interacts with the TMD via its carboxyl group and stabilizes in only one orientation in the binding pocket that does not allow for very strong binding. In contrast, (S)-2,4- DP binds through two moieties simultaneously, a carboxyl group and an aromatic ring with two Cl^−^ groups and stabilizes in several different orientations through hydrophobic interactions that allow stronger binding, resulting in stronger negative allosteric modulation (Nakagita et al., 2019).

These observations provide information about the relevance of structural modification in NAM compounds that could affect their interaction with the receptor. Although TMDs of TAS1R3 are the most likely regions responsible for allosteric modulation, TMDs and VFT regions of TAS1R2 cannot be ruled out completely. For example, the diuretic amiloride binds to TAS1R2 (TMD3, TMD5, TMD7) and inhibits the sweet response in a species dependent manner (Zhao et al., 2018). Further, the umami compound [monosodium glutamate (MSG)] and peptides (Glu-Asp, Glu-Glu) bind to the VFT region of TAS1R2 and inhibit the sweet induced response (Shim et al., 2015). These observations suggest that both subunits are important for allosteric activity of TAS1R2/TAS1R3 and further structural studies are required to design novel sweet allosteric modulators.

## Umami Taste Signal Transduction Mechanisms

In contrast to four well-known basic human tastes (sweet, bitter, salty and sour), umami or ‘savoury taste’ is relatively recent and was introduced in early 2000 by Kikuna Ikeda (Ikeda, 2002) as a new seasoning element in food. The main stimulus for umami taste is the amino acid, L-glutamate present in the diet mainly in the form of MSG (Roper, 2007). Glutamate was first extracted from konbu/kombu (dried kelp of *Fucus vesiculosus*) and described as a “unique taste” and “very different from other tastes”. The terminology “umami” comes from the Japanese word “umai” meaning “delicious.” Moreover, the taste of umami is also produced by food such as mushrooms and soy sauce that contain amino acids (L-aspartate), peptides and synthetic ingredients similar to glutamate and some organic acids (Roper, 2007; Kinnamon, 2009) (Table 5).

**TABLE 5:** Umami receptor agonists with their EC50 values and other pharmacological properties.

Agonist	Nature	EC50 (mM)	Binding pocket	References
L-amino acids (glutamate, aspartate, alanine, serine, asparagine, arginine, histidine, threonine, glutamine)	Amino acids	3 (glutamate), ND for others	VFT (TAS1R1)	(Li et al., 2002; Nelson et al., 2002; Zhang et al., 2008; Toda et al., 2013)
L-theanine	Amino acid (plant origin)	ND	VFT (TAS1R1)	(Narukawa et al., 2014)

VFT, venus flytrap domain; ND, not determined.

The umami receptor (TAS1R1/TAS1R3) is a heteromeric member of the class C GPCRs, whereas most other receptors of this class exist as homodimers (Nelson et al., 2002; Temussi, 2009; Leach and Gregory, 2017). TAS1R1/TAS1R3 is the predominant umami taste receptor (Zhao et al., 2003; Behrens and Meyerhof, 2011) and the TAS1R1 subtype is critical for sensing umami taste as its deletion abolished the response to umami taste stimuli (Mouritsen and Khandelia, 2012). However, TAS1R1/TAS1R3 is not the only receptor capable of detecting umami ligands (Chaudhari et al., 2000; Kunishima et al., 2000; Li et al., 2002; Nelson et al., 2002). Studies using heterologous expression, afferent nerve recordings, and behavioral experiments have confirmed that metabotropic glutamate receptor 1, and 4 (taste-mGluR1 and taste-mGluR4) also sense umami stimuli (Chaudhari et al., 2000; Kunishima et al., 2000; Li et al., 2002; Nelson et al., 2002). Notably, TAS1R3 knock out mice show a strongly diminished response to glutamate and sweet stimuli (Damak et al., 2003) and taste cells isolated from these mice respond to IMP and glutamate which is abolished in presence of mGluR antagonists (Pal Chaudhry et al., 2016). TAS1R1/TAS1R3 is not only activated by glutamate, but this activation is strongly enhanced in the presence of 5′-ribonucleotides, (inosine 5′ monophosphate; IMP) a response that is a hallmark of umami taste (Rifkin and Bartoshuk, 1980).

The main transduction components following the activation of TAS1R1/TAS1R3 are similar to those for sweet taste (Zhang et al., 2003), i.e., α-gustducin (and γ13/β1 or β3), PLCβ2, IP3R and TRPM4/5. Cyclic nucleotides may also contribute to transduction of umami taste in TRCs. When taste tissue is stimulated with umami, its cyclic AMP level is decreased (Abaffy et al., 2003). However, the consequence of decreased cAMP in TRCs has not yet been fully elucidated. Both, α-transducin and α-gustducin are involved in umami taste signal transduction, as mice lacking the gene for one of these proteins showed a reduced response to this taste (He et al., 2004; Leach and Gregory, 2017). In the taste palate fungiform papillae, *α*-gustducin and *α*-transducin activate PDE that reduces cAMP levels. Ligand binding to the TAS1R1/TAS1R3 heterodimer, releases Gβγ subunits to stimulate PLCβ2, which hydrolyzes PIP2 to DAG and IP3 (Kinnamon, 2009). IP3 then activates IP3R3 which results in release of calcium ions from intracellular compartments (Clapp et al., 2001; Leach and Gregory, 2017) (Figure 3). Calcium ions activate TRPM5 and TRPM4 channels, leading to an influx of sodium ions, subsequent cell membrane depolarization, and finally release of ATP, which activates ionotropic purinergic receptors located in sensory fibers (Perez et al., 2002; Sugita, 2006). This pathway was confirmed when mice devoid of TRPM5, TRPM4, PLCβ2, and IP3R3 showed a reduced response to umami taste perception following glutamate stimuli (Damak et al., 2006; Kinnamon, 2009; Eddy et al., 2012).

### Structural, Molecular and Conformational Changes of Umami Receptor

In the last decade, several in depth modeling and mutagenesis approaches have our improved structural and molecular understanding of the umami receptor. The VFT regions of both subunits of TAS1R1/TAS1R3 comprise orthosteric and allosteric ligand binding sites for umami stimuli.

Mutagenesis and molecular modeling studies reveal that the cognate agonist glutamate binds in the VFT region of the TAS1R1 subunit of TAS1R1/TAS1R3 and stabilizes the closed active receptor conformation. Moreover, four residues in the TAS1R1 VFT region (S172, D192, Y220 and E301) showed no detectable response to glutamate when they were mutated to alanine suggesting that they are critical for glutamate binding. The glutamate binding and stabilization of the closed conformation of TAS1R1, activates the downstream signaling pathway, while TAS1R3 remains in an open (inactive) conformation. Therefore, closure of the VFT is the key event that sensitizes umami taste receptor signal transduction (Lopez Cascales et al., 2010). Apart from glutamate, other L amino acids were also found to elicit functional responses by binding to the corresponding VFT region of TAS1R1. Six residues that contributed to the acidic amino acid agonist (L-glutamate and L-alanine) responses have been identified (S148, R151, A170, E174, A302, and D435).

#### Allosteric Modulation of Umami Receptor

Because of significant advancement in understanding and food industry application of umami taste, its allosteric modulators are sought after. Several allosteric umami ligands have been discovered with varying potency, only a few of which have been characterized at the molecular level. The best characterized umami PAMs, the 5′-ribonucleotides: inosine 5′-monophosphate (IMP) and guanosine 5′-monophosphate (GMP), interact with the VFT region of the TAS1R1 subunit to enhance the glutamate induced response that is hallmark of umami taste (Table 6). IMP and GMP binding sites in the VFT are adjacent to that for glutamate binding. Mutation of four residues (H71, R277, S306, and H308) abolished the IMP/GMP induced glutamate response suggesting their involvement in allosteric binding of these nucleotides. Structurally, IMP and GMP stabilize the closed form of the TAS1R1 VFT region through electrostatic interactions and coordinate the positively charged residues that act as pincers. The ability of IMP and GMP to interact with the VFT region (as opposed to the TMD region) represents a unique mechanism of positive allosteric regulation within class C GPCRs (Urwyler, 2011).

**TABLE 6:** Umami receptor allosteric modulators with conc. used in cell based assays and other pharmacological properties.

Allosteric modulators	Nature	Conc. (mM)	Binding pocket	References
IMP/GMP	Nucleotide	1	VFT (TAS1R1)	(Li et al., 2002; Nelson et al., 2002; Zhang et al., 2008)
Cyclamate	Sodium cyclohexylsulfamate	8	TMD (TAS1R3)	(Xu et al., 2004)
Methional (3-methylsulfanylpropanal)	—	0.12	TMD (TAS1R3)	(Toda et al., 2018)
Lactisole (2-4-methoxyphenoxy propionic acid)	Carboyxlic acid salt	5	TMD (TAS1R3)	(Xu et al., 2004)
Clofibric acid (4- chlorophenoxy)-2-methylpropanoic acid	Herbicide acid	4	TMD (TAS1R3)	(Maillet et al., 2009; Kochem and Breslin, 2017)

Where VFT, Venus flytrap domain; TMD, transmembrane domain.

In contrast to IMP and GMP that bind to the TAS1R1 extracellular domain, the well-known flavor compound methional and its analogs bind to the TMD region and allosterically regulate the umami receptor in a species dependent manner (Toda et al., 2018). Importantly, methional utilizes several distinct residues in different TAS1R1 transmembrane domains (TMD2-7) to act as a PAM in the human umami receptor, yet it behaves as a NAM in the mouse counterpart. This unusual phenomenon provided an opportunity to study the mechanisms of both positive and negative modulation in TAS1R1 simultaneously (Toda et al., 2018).

Construction of chimeric receptors between human (h) and mouse (m) and their functional analysis demonstrated that the TMD of TAS1R1 is the key domain for switching the PAM/NAM activities of methional. Point mutation substitutions between these species identified four residues (h/m; F768/L769, N769/H770, S799/T800, and S802/G803) that are collectively required to switch PAM/NAM activities. A similar mode of allosteric regulation and PAM/NAM mode switching has been reported for mGluR5 (Gregory et al., 2013) suggesting this as an unusual and distinct phenomenon of the class C GPCRs. Further, alanine scanning mutagenesis in TAS1R1 of the corresponding residues vital for the activity of other taste inhibitors (sweetener inhibitors; NHDC and cyclamate; sweet and umami taste inhibitor; lactisole) revealed three residues required for PAM (W697^4.50^ F728^5.40^ and F732^5.44^) and a single residue (F642^3.40^) for NAM. These results suggest that both the PAM and NAM activities of methional are conferred by residues that are distinct from those required for the PAM/NAM switch. Knowing that methional is an important part of food seasoning globally, these observations could help in maximizing its use in enhancing flavors along with amino acids and nucleotides.

Despite PAMs being a central focus for umami allosteric modulation, there has also been considerable research on negative allosteric modulation where lactisole emerged as a prominent NAM of the umami receptor, TAS1R2/TAS1R3. Because umami and sweet receptors share the TAS1R3 subunit, findings from studies on sweet receptor lactisole binding are relevant. A comprehensive study on the sweet receptor identified critical residues within the TMD regions (S640^3.32^, H641^3.33^ in TMD3 and F778^6.51^, L782^6.55^ in TMD6) of TAS1R3 required for lactisole binding pocket and showed a large effect on sensitivity to lactisole (Xu et al., 2004; Jiang et al., 2005b). Because lactisole shares structural similarity with two other classes of compound: fibrates and phenoxy-herbicides, researchers studied them to search for novel sweet/umami inhibitors (Maillet et al., 2009). The lipid lowering drug, clofibric acid inhibits the TAS1R3 umami receptor mediated response both *in vitro* and *in vivo* (Table 6). Like lactisole, clofibrate inhibits the umami taste from glutamate by binding with a similar affinity to TAS1R1/TAS1R3. However, its specificity against the umami receptor still needs to be validated alongside other umami taste receptors (mGluR1, mGluR4, or NMDA).

## TYPE 2 TASTE G PROTEIN-COUPLED RECEPTORS (BITTER RECEPTORS)

Type 2 taste GPCRs are represented by bitter taste receptors that have a distinct subset of bitter sensing cells in type II TRCs and notably 25 bitter taste receptors (TAS2Rs) are reported to be expressed in humans (Chandrashekar et al., 2000; Devillier et al., 2015; Behrens and Meyerhof, 2018). A significant amount of work has been done to explore the diversity among TAS2Rs and their agonists in taste biology (Adler et al., 2000; Behrens and Meyerhof, 2009; Behrens and Meyerhof, 2018). Some TAS2Rs (TAS2R3, TAS2R5, TAS2R13, TAS2R50) are narrowly tuned to structurally similar bitter compounds, whereas others are broadly tuned (TAS2R10, TAS214, TAS2R46), responding to several bitter compounds. Initially it was believed that each bitter-sensitive type II TRC expressed every TAS2R isoform (Adler et al., 2000) but other studies suggest that TAS2Rs can be expressed differentially, allowing for possible discrimination among bitter compounds (Caicedo and Roper, 2001; Behrens and Meyerhof, 2009; Behrens et al., 2009). Please refer to figure 4B for the basic structure of the bitter receptor.

### Bitter Taste Signal Transduction Mechanisms

Bitter taste is the most complex of all the five basic tastes and provides protection against ingestion of toxic substances by eliciting an innate aversive response across species (Chandrashekar et al., 2006; Behrens and Meyerhof, 2018). The TAS2Rs that mediate bitter taste perception are among ∼50 TAS2Rs identified in mammals, and 25 known to be expressed in humans (Adler et al., 2000; Devillier et al., 2015; Yoshida et al., 2018). TAS2R family is the most diverse and binds to a wide range of agonists compared with the other taste GPCRs (Jaggupilli et al., 2016) (Supplementary Table 1).

TAS2Rs are distinctive among class A GPCRs in that many of them bind agonist with low apparent affinity in the micromolar range, rather than the nanomolar range (Di Pizio et al., 2016). The activation of TAS2Rs by harmless, minute amounts of bitter compounds such as those contained in most vegetables would limit the availability of food resources appearing safe for consumption and therefore could negatively affect survival. Hence, the concentration ranges at which bitter taste receptors are activated are well-balanced to allow species to maintain a healthy diet yet avoid ingestion of spoiled food containing strongly bitter ligand.

Hundreds of bitter compounds have been reported to evoke bitterness and activate human bitter receptors in different cell based assays. These bitter agonists include plant-derived and synthetic compounds such as peptides, alkaloids and many other substances (Supplementary Table 1). (Pronin et al., 2004; Meyerhof et al., 2010; Iwata et al., 2014). Some TAS2Rs are activated by a wide range of compounds, whereas others show strict specificity for a single bitter compound (Behrens et al., 2009; Sakurai et al., 2010a; Born et al., 2013). Interestingly, TAS2R31, TAS2R43, and TAS2R46 have around 85% sequence homology, but they bind to different agonists (Brockhoff et al., 2010; Jaggupilli et al., 2016), reinforcing the idea that each TAS2R might have a unique ligand-binding pocket.

The canonical TAS2R signal transduction cascade signaling molecules shared among bitter sweet and umami receptors (Wong et al., 1996; Huang et al., 1999; Mueller et al., 2005), include the heterotrimeric G protein subunits (Gα-gustducin, Gβ3, and Gγ13), (Ishimaru, 2009; Shi and Zhang, 2009), a phospholipase C (PLCβ2), an inositol trisphosphate receptor (InsP3R), and the TRPM5 ion channel. Upon receptor activation by bitter ligands the G protein α-gustducin dissociates from its βγ subunits. The latter activates PLCβ2, leading to a release of Ca^2+^ from IP3-sensitive Ca^2+^ stores, resulting in Na^+^ influx through TRPM5 channels. This Na^+^ influx depolarizes the cells and causes the release of neurotransmitter ATP through gap junction hemichannels or CALHM1 ion channels (Finger et al., 2005; Chaudhari and Roper, 2010; Taruno et al., 2013) (Figure 3).

### Structural, Molecular and Conformational Changes of Bitter Receptors

Classification of TAS2Rs has always been ambiguous because they were originally considered to be a distinct family (Horn et al., 2003) or grouped with the frizzled receptors (Fredriksson et al., 2003; Jaggupilli et al., 2016), but most recent analyses (Di Pizio et al., 2016) support their classification with Class A GPCRs. The ability of bitter taste receptors to interact with numerous structurally diverse substances compared to other GPCRs is remarkable and includes a wide range of drugs/antibiotics, polyphenols, bacterial metabolites, salts and metal ions (Supplementary Table 1). Therefore, exploring the criteria for identification of highly heterogeneous bitter compounds with pronounced selectivity has become a major research area. Some of these studies rely solely on in silico homology/computational modeling (Dai et al., 2011; Tan et al., 2012; Di Pizio et al., 2020; Dunkel et al., 2020) and others on *in vitro* genetic modification and functional assay systems (Pronin et al., 2004; Nowak et al., 2018; Jaggupilli et al., 2019).

As a group of over ∼50 receptor subtypes, TAS2Rs recognize structurally diverse agonists where some are broadly tuned (TAS2R46, TAS2R14, TAS2R10, and TAS2R43) recognizing diverse agonists, while others (TAS2R1, TAS2R4, TAS2R7) show strong selectivity and narrow tuning (Liu et al., 2018; Wang et al., 2019). The agonist binding cavity in most bitter GPCRs is located deep within their transmembrane domain (TMD), with the exception of TAS2R7 in which it resides on the extracellular surface (Liu et al., 2018). TAS2Rs are also distinct in containing highly conserved TMD regions, with thirteen key residues and two motifs (LXXXR in TMD2 and LXXSL in TMD5) that are absent in class A GPCRs, and may reflect their different activation mechanisms (Singh et al., 2011). LXXSL plays a structural role by stabilizing the helical conformation of TMD5 at the cytoplasmic end and a functional role by interacting with residues in intracellular loop 3 (ICL3) which is important for proper receptor folding and function (Singh et al., 2011). Moreover, mutation of the conserved residues in LXXSL and LXXXR motifs results in protein misfolding and poor surface expression (Singh et al., 2011; Pydi et al., 2014a).

The initial study highlighting the structure–activity relationship of bitter taste receptors was performed with receptors belonging to a subfamily of closely related TAS2Rs (Pronin et al., 2004). By physically swapping the extracellular loop 1 (ECL1) between TAS2R43 and TAS2R31, chimeric TAS2R31/TAS2R43 (ECL) gained responsiveness to the compound n-isopropyl-2methyl-5-nitrobenzenesulfonamide (IMNB), whereas the reverse chimera TAS2R31 (ECL)/TAS2R43 lost responsiveness for IMNB. Although this report supports an important contribution of residues located within the transmembrane region of the investigated receptors, the extracellular loops appear to be of importance for agonist selectivity. This empirical finding contrasts with earlier computational studies which predicted the agonist binding site to lie within the helical bundle of TAS2Rs without particular contacts between extracellular loops and docked agonists (Floriano et al., 2006; Miguet et al., 2006).

#### Bitter Receptor Ligand Binding Pocket

The emergence of TAS2Rs as the most broadly tuned taste receptors might give the impression that their specific interaction with numerous agonists is because of several binding pockets that accommodate subgroups of bitter compounds. However, structure–function analysis of TAS2Rs (except for TAS2R7) has demonstrated the presence of only a single agonist binding pocket comprising the upper parts of TMD2, TMD3, TMD5, TMD6 and TMD7. The reason for their broad tuning and recognition of such a broad spectrum of agonist might most likely be attributed to the presence of an additional extracellular binding site called a “vestibular site,” in addition to the orthosteric selecting as reported for TAS2R46 (Sandal et al., 2015). This two site architecture offers more ligand recognition points than a single one, and thus might help in selecting the appropriate agonists. Moreover, the presence of the vestibular site may also help to discriminate among the wide spectrum of bitter ligands.

Although broadly tuned receptors (TAS2R46, TAS2R31 and TAS2R43) have high homology in amino acid sequence, their agonist profiles only slightly overlap (Kuhn et al., 2004; Brockhoff et al., 2007; Di Pizio and Niv, 2015) which suggests the involvement of key residues at different positions in agonist specificity. Consequently, when strychinine interacting positions in TAS2R46 (residues differ at this position in TAS2R31, TAS2R43) were exchanged between these two receptors not only was the strychnine responsiveness transferred to the recipient receptor (TAS2R31, TAS2R43), but also sensitivity to additional TAS2R46 agonists (absinthin and dentaonium). Sensitivity to activation by aristolochic acid was lost in the mutant receptors (Brockhoff et al., 2010). This experimental evidence supports the presence of a common agonist binding pocket and agrees with other studies on TAS2R16, TAS2R14 and TAS2R7 receptors (Sakurai et al., 2010a; Sakurai et al., 2010b; Thomas et al., 2017; Liu et al., 2018; Nowak et al., 2018).

Recent studies used homology modeling and mutagenesis to elucidate the nature of the ligand binding pocket in TAS2R7, TAS2R14 and TAS2R16 receptors (Thomas et al., 2017; Liu et al., 2018; Nowak et al., 2018). They reported that the binding pocket is flexible and wide open to accommodate molecules of diverse size and shape, and thus permits chemical modifications among agonists as well (Thomas et al., 2017; Liu et al., 2018; Nowak et al., 2018). Although the molecular basis for the promiscuity of bitter receptors is attributed to their apparent flexible spacious binding site, future work elucidating the contact points between TAS2Rs binding site residues and its agonists in terms of additional binding locations is required.

#### Bitter Receptors Ligand Binding Domain and Amino Acid Residues

A majority of the TAS2R studies based on molecular modeling, mutagenesis and heterologous expression systems (Biarnes et al., 2010; Brockhoff et al., 2010; Tan et al., 2012; Nowak et al., 2018; Shaik et al., 2019) suggest that the ligand binding pocket is formed by several key residues in most TMDs (TMD1, TMD2, TMD3, TMD5, TMD6 and TMD7), with the exception of TMD4.

Studies show similarities as well as differences regarding residues and positions involved in agonist-receptor interactions. However, most of them agree that besides position N^3.36^ in TMD3 (superscript as per Ballestros-Weinstein nomenclature for class A GPCRs) (Ballesteros and Weinstein, 1995) and other residues (L^3.32^, L^3.33^, and E^3.37^) in its close proximity, play a role in agonist activation of several broadly tuned TAS2Rs (TAS2R1, TAS2R16, TAS2R30, TAS2R38, TAS2R46) (Pronin et al., 2004; Biarnes et al., 2010; Brockhoff et al., 2010; Sakurai et al., 2010b; Dai et al., 2011). In contrast, for the narrowly tuned TAS2R7, one position in TMD3 (H94^3.37^) and another in TMD7 (E264^7.32^) were found crucial for metal ion binding (Wang et al., 2019). Mutagenesis and molecular modeling revealed that these two residues contribute to the metal ion binding pocket in TAS2R7. Moreover, metal ions bind distinctively to residues lining the binding pocket and interestingly, the presence of calcium in the assay solution appears to affect the TAS2R7 response to metal ions. It is not clear how calcium affects metal ion binding to TAS2R7, but it might work cooperatively with certain ions and not others. Future studies focusing on structural interactions between the receptor and metal ions will provide further insights into how they activate the receptor.

In TMD2, two studies suggest that position N^2.61^ is critical for binding in TAS2R1 (Singh et al., 2011) and TAS2R46 (Brockhoff et al., 2010). Likewise, in TMD7, position 265^7.39^ is implicated in binding to TAS2R46 (E265) and TAS2R1 (I263) (Dai et al., 2011). In TMD5, position H^5.43^ is implicated in binding in TAS2R16 and E^5.46^ in TAS2R1 (Dai et al., 2011) while, in TMD7, position E^7.32^ was crucial for metal ion binding (Wang et al., 2019). These residues represent putative contact points for agonist interaction and form a pattern in being spaced one helical turn from each other.

Recent mutagenesis studies (Nowak et al., 2018; Di Pizio et al., 2020) performed in broadly tuned TAS2R14 with agonists (aristolochic acid, picrotoxinin, thujone) found several residues in TMDs to be involved in agonist binding. However, in contrast to TAS2R10 (Born et al., 2013) and TAS2R46 (Brockhoff et al., 2007), mutation of TAS2R14 did not result in complete loss of function for all agonists but a varied reduction in responsiveness or selectivity toward agonists. Among several mutants, only mutation of W89A resulted in complete loss of responsiveness against picrotoxinin while others showed more subtle agonist selective changes. This indicates that TAS2R14 is not streamlined for the most sensitive detection of selected agonists, but rather tailored to detect numerous diverse agonists, with comparatively lower apparent affinity.

The binding characteristics of bacterial acyl homoserine lactones (AHLs) on TAS2Rs (TAS2R4, TAS2R14 and TAS2R20) suggest the presence of a single orthosteric site situated close to the extracellular surface and reinforce the significant role of the extracellular loop structure (ECL2) in TAS2R ligand binding and activation (Jaggupilli et al., 2018). The crucial AHL binding residues in TAS2R4 and TAS2R14 are predominantly located in the ECL2, while in TAS2R20 they are present in TMD3 and TMD7 helices. The ECL2 residues, N165 in TAS2R4, and R160 and K163 in TAS2R14 were found crucial for lactone binding. In contrast, TAS2R20 residues W88 (TMD3) and Q265 (TMD7) are essential for agonist binding (Pydi et al., 2014c; Zhang et al., 2017; Jaggupilli et al., 2018). In addition, the hydrophobic amino acids in the three TAS2Rs are considered important in directing the orientation of the hydrophobic acyl chains of lactones that facilitate receptor activation.

The transmembrane domain in GPCRs is composed mainly of hydrophobic amino acids accommodated in the plasma membrane. Therefore, hydrophobic properties of the receptor binding pocket are important for any membrane accessible agonist. Hydrophobic residues in TMD3 and TMD7 of TAS2R16 are important in forming a wide ligand-binding pocket (Thomas et al., 2017) that accommodates larger ligands like the β-glycosides. By using salicin analogs as TAS2R16 novel agonists (differ structurally to salicin in β-glucoside core constituents), several critical residues were identified that are required for signaling. Interestingly, these were identical to the residues critical for salicin signaling, except for W261, which was not required for activation by the analog 4-NP-β-mannoside. Importantly, all these residues are in the TMD helices or intracellular face of the receptor, consistent with classical GPCR signal transduction. These results suggest that larger ligands bind to the wide binding pocket of TAS2R16 on the extracellular side, and then their signal is transduced via conserved residues on the intracellular side. This can account for the broad spectrum of ligand recognition conferred by TAS2R16.

Unlike broadly tuned receptors, narrowly tuned ones like TAS2R7 show two different types of critical residue in ligand binding. The first type includes D86, W170 and S181 that are agonist independent and their mutation significantly reduces the ability of TAS2R7 to bind agonist, while a second group consisting of D65 and W89 are selective for quinine and enhance binding to a specific category of ligand (Liu et al., 2018).

Despite the variation in the amino acid type and location important for agonist binding among receptors of the bitter family, for the most part, ligand binding pockets are present on the extracellular surface of TMDs or on ECL2. The function of the residues at these binding pockets is dictated by multiple factors that include the type of ligand, the movements in TMDs, and the associated movement of ECL2 to accommodate the ligand. Structure–function studies have identified a conserved KLK/R motif in the intracellular carboxyl terminal domain of 19 TAS2Rs that is critical for cell surface expression, trafficking and receptor activation (Upadhyaya et al., 2015; Jaggupilli et al., 2016).

#### Agonist, Antagonist Binding and Modulation of Bitter Receptors

In simple pharmacological terms an antagonist is a ligand that inhibits the biological response induced by an agonist and does not induce any response of its own, while a ligand that reduces the constitutive/basal activity of a GPCR is considered an inverse agonist. An antagonist acts as a competitive inhibitor to block receptor activity. Large numbers of agonists have been identified for bitter receptors, but few antagonists have been found so far (Table 7). Finding an antagonist/inhibitor for bitter taste would not only help in understanding the TAS2R mechanism of signal transduction but have potential use in foods to overcome unwanted bitterness in consumer products. Such bitter blockers have been proposed to increase the palatability of bitter tasting food and beverages, increase the compliance in taking bitter tasting drugs, especially children’s formulations and reduce or prevent off-target drug effects in extra-oral tissues (Clark et al., 2012).

**TABLE 7:** Bitter taste receptor inhibitors with their IC50 values and other pharmacological properties.

Antagonist	Mode of action	Bitter receptors	Tested agonists	IC50 (µM)	References
GIV3727or 4-(2,2,3-trimethylcyclopentyl) butanoic acid	Competitive orthosteric inhibitor	31	acesulfameK	6.4	(Slack et al., 2010)
		43	Aristolochic acid	11.33	
		4	Cochicine	108	
		40	Cohumulone	6.24	
Gamma-aminobutyric acid (GABA)	Orthosteric inhibitor	4	Quinine	3.2	(Pydi et al., 2014b)
3β-hydroxydihydrocostunolide (3HDC)	ND	46	Absinthin	14.1	(Slack et al., 2010; Brockhoff et al., 2011)
			Andrographolide	4.9	
			Denatonium	6.8	
			Picrotoxinin	4.7	
			Strychnine	15.3	
3-hydroxypelenolide(3HP)	ND		Absinthin	57.8	(Brockhoff et al., 2011)
			Andrographolide	44.5	
			Denatonium	51.4	
			Picrotoxinin	22.9	
			Strychnine	84.9	
Probenecid	Allosteric inhibitor	16	Salicin	292	(Greene et al., 2011)
Sakuranetin	ND	31	Saccharin	5.5	(Fletcher et al., 2011)
6-Methoxysakuranetin	ND	31	Saccharin	10.2	(Fletcher et al., 2011)
Jaceosidin	ND	31	Saccharin	11.7	(Fletcher et al., 2011)
6,3′-dimethoxyflavanone	ND	39	Epicatechin gallate (ECG)	4075	(Roland et al., 2014)
			Denatonium	240	
6-Methoxyflavanone	ND	39	Epicatechin gallate (ECG)	479	(Roland et al., 2014
N,N-bis(carboxymethyl)-l-lysine(BCML)	ND	4	Quinine	0.059	(Pydi et al., 2014b)
(±) abscisic acid (ABA)	ND	4	Quinine	34.4	(Pydi et al., 2015)

ND, not determined.

To date ∼12 bitter inhibitors have been reported to interact with only 10 TAS2Rs subtypes (Table 5) by binding to transmembrane domains in a similar manner to agonist. GIV3727 (4-(2,2,3-trimethylcyclopentyl) butanoic acid) was the first TAS2R antagonist discovered and to be well characterized structurally (Slack et al., 2010) that acts as an orthosteric competitive antagonist for TAS2R31. It competes with the acesulfame K agonist both *in vitro* and *in vivo*. GIV3727 is moderately selective because it inhibits multiple bitter receptors including, TAS2R4, TAS2R40 and TAS2R43. Homology modeling revealed that the -COOH group in GIV3727 is important for ligand-receptor interactions as its replacement with an ester or the corresponding alcohol abolished its antagonist activity. Moreover, a mutagenesis study in TAS2R31 and TAS2R43 revealed residues K265^7.39^ and R268^7.39^ in TMD7 to be crucial for its antagonistic activity (Slack et al., 2010). Similarly, another non-selective inhibitor, probenecid (p-(dipropylsulfamoyl) benzoic acid) was found to act as NAM of TAS2R16 activity and inhibits TAS2R38 and TAS2R43 as well (Greene et al., 2011). Two point mutations, P44T and N96T in TMD3 of hTAS2R16 were found to significantly suppress the ability of probenecid to inhibit salicin activity. Hydrophobicity seems important for their pharmacological activity as observed for both probenecid and GIV3727. The sesquiterpene lactone, 3β-hydroxydihydrocostunolide (3HDC) is an interesting bitter blocker as it acts as a competitive antagonist of TAS2R46, TAS2R30, TAS2R40, yet activates TAS2R4, TAS2R10, TAS2R14 and TAS2R31 as an agonist (Brockhoff et al., 2011).

Similarly, various flavonones were also noted as antagonists for TAS2R31, TAS2R39 with varying efficacy. Taken together most of the currently known antagonists are non-selective and there is an urgent need for studies that focus on selective antagonists of major broadly tuned TAS2Rs (such as TAS2R10, TAS2R14, TAS2R16 and TAS2R46). In order to target bitterness in terms of food industry needs, potential peptide inhibitors from different protein sources such as hen protein hydrolysates (inhibits TAS2R4, TAS2R7, TAS2R14) and beef proteins (inhibits TAS2R4) (Zhang et al., 2018; Xu et al., 2019) are reported to be effective. Several umami glutamyl peptides isolated from soyabean have been found to act as non-competitive allosteric inhibitors of TAS2R16 against the salicin induced response (Kim et al., 2015).

#### Constitutive Activity of Bitter Receptors

A phenomenon in GPCR activity is that of constitutive activity, essentially an active state occurring in the absence of agonist which has been demonstrated in more than 60 GPCRs (Seifert and Wenzel-Seifert, 2002). It is the production of a second messenger or downstream signaling by a receptor in ligand independent manner. Constitutive activity provides another possibility for taste inhibitor discovery using inverse agonists. Inverse agonists can inhibit both agonist-dependent and agonist-independent activity, while antagonists can inhibit only agonist-dependent activity (Chalmers and Behan, 2002). Interestingly, some mutations in GPCRs can lead to constitutive activity and receptors with this characteristic (including constitutively active mutants or CAM) are important tools to investigate new bitter inhibitors. Although constitutive activity has not been observed naturally in TAS2Rs, when induced by mutation these receptors provide a useful means to investigate the relationship between an active receptor conformation and inverse agonist pharmacology.

Molecular modeling and functional assays report five CAMs critical residues for TAS2Rs, one in TMD7 (S285^7.47^) and four others in intracellular loop 3 (H214A, Q216A, V234A, and M237A) (Pydi et al., 2014a; Pydi et al., 2014b). Of the five CAMs, only the TAS2R4 with H214A mutation shows a 10 fold increase in constitutive activity. This histidine residue is highly conserved in most TAS2Rs. Mutation of H214 (H214A) helped in finding two new inverse agonists (GABA and ABA; Table 7) (Pydi et al., 2015). Similar pharmacological approaches can be used to generate mutants of all TAS2Rs to screen for their inverse agonist/bitter taste blockers. However, for better characterization and interpretation of TAS2Rs, future *in vivo* studies should be performed to understand the functional relevance of these CAMs. At the same time, it is worth noting that the potential presence of endogenous agonists makes it difficult to determine the true constitutive activity of GPCRs including TAS2Rs (Devillier et al., 2015).

## Kokumi Sensation Signal Transduction

In addition to the five basic tastes, sensations beyond these add another dimension to taste perception. One such example is “kokumi” that is distinct from the other five tastes in that it does not have a taste as such but rather induces a sensation of “mouthfulness,” depth, thickness and aftertaste in the flavors. Although, this flavor has been used historically and is well recognized in Japanese cuisine, it was first characterized by Ueda et al. (1990) who isolated a kokumi taste substance from water extracts of garlic and onion and identified, γ-glutamylcysteinylglycine or glutathione (GSH) as the main active ingredient of kokumi flavor (Ueda et al., 1990; Ueda et al., 1997; Dunkel et al., 2007). GSH is abundantly present in food-grade yeast extract and has been used to make foods more flavoursome.

Kokumi signal transduction was unknown until CaSR expression was reported in a subpopulation of taste cells in mice and rats that suggested it could function as a taste receptor for calcium and amino acids (San Gabriel et al., 2009; Bystrova et al., 2010). However, its apparent role in kokumi stimuli detection was not confirmed. Ohsu et al. (2010) for the first time reported that kokumi peptides (GSH, γ-Glu-Val-Gly and various γ-glutamyl peptides; Table 8) signal through CaSR and can synergise with sweet, salty, and umami taste qualities to impart an augmented kokumi sensation, i.e., increased depth of flavor which was further complemented by later studies (Maruyama et al., 2012; Kuroda and Miyamura, 2015). By using heterologous expression systems and human sensory analysis these studies demonstrated that kokumi peptides impart kokumi sensation to sweet, salty and umami taste via CaSR as the kokumi component was specifically suppressed in the presence of the CaSR-specific NAM NPS-2143. To further validate this idea, Maruyama et al. (2012) identified a distinct population of taste cells expressing CaSR in mouse lingual tissue which did not express either sweet or umami receptors. Notably, these cells are specifically responsive to kokumi substances and elicit a Ca^2+^ response to focally applied kokumi stimuli in mouse lingual slices. Moreover, this response was inhibited in the presence of NPS-2143. These findings support the idea that CaSR mediates kokumi sensation effects in TRCs.

**TABLE 8:** Kokumi sensation receptor agonists, allosteric modulators with concentrations used in cell based assays.

Agonist	Type/nature	Conc. (mM unless stated otherwise)	Binding pocket	References
Ca^2+^	Orthosteric agonist/cation	1^a^	VFT	(Brown et al., 1993; Conigrave et al., 2000; Breitwieser, 2006)
Mg^2+^	Orthosteric agonist/cation	10^a^	VFT	(Brown et al., 1993)
Gd^3+^	Orthosteric agonist/cation	0.02^a^	VFT	(Brown et al., 1993)
Al^3+^	Orthosteric agonist/cation	0.5	VFT	(Brown et al., 1993)
Sr^2+^	Orthosteric agonist/cation	0.5	VFT	(Brown et al., 1993)
Mn^2+^	Orthosteric agonist/cation	0.5	VFT	(Brown et al., 1993)
Ni^2+^	Orthosteric agonist/cation	0.5	VFT	(Brown et al., 1993)
Ba^2+^	Orthosteric agonist/cation	0.2	VFT	(Brown et al., 1993)
Spermine	Orthosteric agonist/polyamine	0.15^a^	VFT	Quinn et al., 1997)
Spermidine	Orthosteric agonist/polyamine	0.002^a^	VFT	(Nemeth et al., 2018)
Neomycin	Orthosteric agonist/aminoglycoside antibiotic	0.06^a^	VFT	(Katz et al., 1992)
Gentamicin	Orthosteric agonist/aminoglycoside antibiotic	0.15^a^	VFT	Katz et al., 1992)
Kanamycin	Orthosteric agonist/aminoglycoside antibiotic	0.1	VFT	(Katz et al., 1992)
Amyloid β-peptides	Orthosteric agonist/Peptide	0.001–0.04	—	(Ye et al., 1997)
Poly-Lysine	Orthosteric agonist/peptide	0.03 µM^a^	VFT	(Brown et al., 1991; Nemeth et al., 2018)
Poly L-arginine	Orthosteric agonist/peptide	0.004 µM^a^	VFT	Brown et al., 1991; Nemeth et al., 2018)
Lysozyme	Agonist/protein	0.59^a^	ND	(Yamamoto et al., 2020)
Thaumatin	Agonist/protein	0.07^a^	ND	(Yamamoto et al., 2020)
Aromatic L-amino acids (Trp, Phe, His, Ala, Ser)	PAMs	10	VFT	(Conigrave et al., 2000; Mun et al., 2004; Geng et al., 2016)
Anions (SO_4_^2-^)	NAM	10	VFT	(Geng et al., 2016)
Cinacalcet	PAM/phenylalkylamine	0.051 µM^a^	TMD	(Miedlich et al., 2002; Petrel et al., 2004; Nemeth et al., 2004)
Calindol	PAM/phenylalkylamine	0.31 µM^a^	TMD	Miedlich et al., 2002; Petrel et al., 2004)
NPS R-568	PAM/phenylalkylamine	0.5 µM^a^	TMD	(Miedlich et al., 2002; Petrel et al., 2004)
NPS R-467	PAM/phenylalkylamine	0.01	TMD	(Miedlich et al., 2002; Petrel et al., 2004)
γ-Glu-Val-Gly	PAM/Peptide	0.041 µM^a^	—	(Ohsu et al., 2010)
γ-Glu-Cys-Gly (Glutathione)	PAM/Peptide	76.5 µM^a^	VFT	(Ohsu et al., 2010; Wang et al., 2006
γ-Glu-Ala	PAM/Peptide	3.65 µM^a^	ND	(Wang et al., 2006; Ohsu et al., 2010)
γ -Glu-Val	PAM/Peptide	1.34 µM^a^	ND	(Wang et al., 2006; Ohsu et al., 2010)
γ -Glu-Cys	PAM/Peptide	0.45 µM^a^	VFT	(Ohsu et al., 2010; Wang et al., 2006)
γ -Glu-α-aminobutyryl-Gly (Opthalmic acid)	PAM/Peptide	0.018 µM^a^	ND	(Ohsu et al., 2010)
NPS2143	NAM	0.0003 (IC50)	TMD	(Gowen et al., 2000; Petrel et al., 2004)
Calhex 231	Mixed PAM/NAM	0.1–1 µM (PAM); 3–10 µM (NAM)	TMD	(Petrel et al., 2003; Petrel et al., 2004; Gregory et al., 2018)

Where VFT, venus flytrap domain; TMD, transmembrane domain; ND, not determined.

a shows EC50 value.

More recently, kokumi peptides have been found to have an extraoral physiological role in the gastrointestinal tract where they stimulate secretion of hormones (cholecystokinin and glucagon-like peptide1 by activating CaSR (Depoortere, 2014; Yang et al., 2019). However, future studies with tissue specific deletion of CaSR in taste buds would be helpful in delineating its role in taste physiology.

CaSR involvement in taste is a relatively recent discovery, but its central role in extracellular calcium homeostasis in mammals is well recognised (Brown et al., 1993; Brown, 2013). Diverse ligands activate CaSR, including cations (Ca^2+^ and Gd^3+^), peptides, polyamines (Brown and MacLeod, 2001) and amino acids (Conigrave et al., 2000; Conigrave and Hampson, 2006) (Table 8). Unlike other taste modalities (sweet, bitter and umami), CaSR–ligand binding and recruitment of G protein results in the activation of an intricate, amplifying signaling network which initiates numerous intracellular functions. The functional diversity of CaSR results from its ability to activate multiple Gα proteins (Gq/11, Gi/o, G12/13 and Gs) (Magno et al., 2011; Conigrave and Ward, 2013) which subsequently affect multiple signaling pathways related to the pathophysiology of parathyroid hormone secretion, cancer and metastasis (Kelly et al., 2007; Wettschureck et al., 2007; Mamillapalli et al., 2008).

Kokumi substrates activate CaSR and transmit their signal through Gαq/11 proteins which further activate PLCβ that results in release of intracellular Ca^2+^ store through activation of IP3 receptor channels in the ER. Whether the kokumi pathway strictly relies on Gαq/11 protein or can also use Gα-gustducin, like other taste modalities for downstream signaling, is still unknown (Figure 3). The growing number of reports on kokumi flavor signal transduction are shedding light on its potential use as a flavor enhancer.

### Structural, Molecular and Conformational Changes of Kokumi Receptor

CaSR belongs to the class C GPCR. Within this class, CaSR and metabotropic glutamate receptors (mGluRs) are known to function as disulfide-linked homodimers (Bai et al., 1998; Ward et al., 1998; Pidasheva et al., 2006) (Figure 4A). Structurally, the human CaSR is similar to sweet and umami taste receptors but differs in being a homodimer instead of a heterodimer (Hendy et al., 2013). The ECD of CaSR not only senses nutrients (Ca^2+^, L-Phe and polypeptides; Table 8) and allows ligand to modulate CaSR cooperatively, but is also required for its dimerization (Ray et al., 1999; Zhang et al., 2014). Binding of Ca^2+^ and other ligands to the ECD changes the conformation of the seven transmembrane domains, causing alterations in the intracellular loops and the intracellular domain (ICD), which further trigger downstream signaling pathways (Brown et al., 1975). The ICD is relatively diverse among species and participates in controlling CaSR signaling in multiple ways by modulating receptor expression, trafficking and desensitization (Gama and Breitwieser, 1998; Ward, 2004; Huang et al., 2006).

Homology modeling, mutagenesis and heterologous expression revealed distinct and closely located binding sites for Ca^2+^ and aromatic L-amino acids, in VFT and the cleft of the VFT, respectively (Silve et al., Conigrave et al.,2000; Huang et al., 2009). Notably, four putative Ca^2+^ binding sites of varying affinity have been predicted in the VFT of the CaSR and in which the interaction between site 1 and the other three sites plays a central role in positive cooperativity in sensing Ca^2+^ (Zhang et al., 2014). Besides Ca^2+^, aromatic L amino acids (L-Trp, L-Phe) also activate the CaSR by binding adjacent to the VFT region through three serine and one threonine residue (S169/S170/S171/T145). Interestingly, the double mutation T145/S170 was found to selectively impair L amino acid (Phe, Trp, His) sensing of CaSR, while Ca^2+^ sensing remained intact (Mun et al., 2004; Mun et al., 2005).

The recent crystal structure of the entire extracellular domain of CaSR (Geng et al., 2016) identified four novel Ca^2+^ binding sites in each protomer of the homodimer including one at the homodimer interface which does not correspond to any of the sites reported previously by Huang et al., (2007). It is unclear why these additional calcium binding sites were not found in earlier studies. This might be due to the different expression systems used, crytallization conditions and methods of analysis. The conditions of the more recent studies may have stabilised an active conformational state in which these calcium sites become available (Geng et al., 2016). Among these four Ca^2+^-binding sites, site 4 seems most relevant to receptor activation as it directly participates in the active CaSR conformation. Moreover, a previously reported natural mutation G557E (Hendy et al., 2009) reduced the potency of Ca^2+^ possibly by affecting backbone conformation, thereby weakening the affinity of Ca^2+^ for this site. This confirms that a Ca^2+^ ion at site 4 stabilizes the active conformation of the receptor by facilitating homodimer interactions between the membrane proximal LBD2 region and CRD of CaSR.

The most interesting aspect of Ca^2+^ and L-amino acid interplay was reported by Zhang et al. (2014) who studied L-Phe binding characteristics by monitoring intracellular [Ca^2+^]_i_ oscillations in living cells and performing molecular dynamic simulations. Their findings supported a previous observation that the L-Phe binding pocket is adjacent to the Ca^2+^ binding site 1. Importantly, by binding to this site, L-Phe influences all Ca^2+^ binding sites in the VFT region and enhances CaSR functional cooperativity through positive heterotropic cooperativity to Ca^2+^. Moreover, the dynamic communication of L-Phe at its predicted binding site in the hinge region with the Ca^2+^ binding sites not only influences the adjacent Ca^2+^ binding site 1, but also globally enhances cooperative activation of the receptor in response to alterations in extracellular Ca^2+^.

The crystal structures (Geng et al., 2016) of the entire ECD region of CaSR in the resting and active conformations have provided additional information about the dynamics between calcium and L-amino acid binding (Geng et al., 2016). Most importantly, by using L-Trp, the study provided direct evidence that L-amino acids are CaSR co-agonists, and they act concertedly with Ca^2+^ to achieve full receptor activation. Several lines of evidence support this contention: 1) L-Trp binds at the interdomain cleft of the VFT, which is a canonical agonist-binding site for class C GPCRs (Kunishima et al., 2000; Muto et al., 2007; Geng et al., 2016) and shares a common receptor-binding mode with the endogenous agonists (amino acids or their analogs) of mGluR and GABA_B_ receptors, (Kunishima et al., 2000; Tsuchiya et al., 2002; Muto et al., 2007; Geng et al., 2016). 2) L-Trp interacts with both LBD1 and LBD2 in ECD to facilitate its closure, a crucial first step during CaSR activation. In contrast, no Ca^2+^ ion is found at the putative orthosteric agonist-binding site to induce domain closure. 3) Mutations of L-Trp-binding residues (S147A, S170A, Y218A, and E297K) severely reduced Ca^2+^ induced IP accumulation and intracellular Ca^2+^ mobilization (Zhang et al., 2002; Silve et al., 2005), indicating that L-Trp is required for a Ca^2+^ induced receptor response. Notably, the presence of extracellular Ca^2+^ above a threshold level is required for amino-acid-mediated CaSR activation, amino acids increase the sensitivity of the receptor toward Ca^2+^. Taken together, amino acids and Ca^2+^ ions act jointly to trigger CaSR activation.

Knowing that aromatic L-amino acids (Trp, Phe, His) are important tastants in kokumi flavor, CaSR becomes more relevant for taste biology. Moreover, the kokumi tripeptide, glutathione (GSH) and glutamyl peptide are suggested to bind allosterically to CaSR at the same site as L-amino acids (Wang et al., 2006; Broadhead et al., 2011) and enhance its activity in the presence of 0.5–1 mM free calcium, thereby acting as a positive allosteric modulator. In addition, an ECD crystal structure might help to explain structural and molecular details of the GSH binding pocket such as the nature of critical residues and their binding characteristics. In view of recent reports of calcium emerging as taste modifier, it would be worth investigating how GSH and Ca^2+^ operate in kokumi human perception.

### Allosteric Modulation of Calcium-Sensing Receptor

Classically CaSR is known to be involved in pathophysiology of parathyroid and renal related diseases by sensing calcium ions in extracellular fluid (Brown, 2007). Research on related therapeutic applications has identified several classes of PAMs and NAMs that modulate CaSR agonist sensitivity. More recently this has been applied to kokumi taste signal transduction.

#### Endogenous Modulators (L-amino Acids, Anions and Glutathione Analogs)

Several studies based on molecular modeling and mutagenesis report L-amino acids (L-Phe, L-Tyr, L-His and L -Trp) as PAMs because they enhance the Ca^2+^ induced response of CaSR. Aromatic L-amino acids bind in the VFT domain (Mun et al., 2004) and require a highly conserved five residue binding motif (S147, S170, D190, Y218 and E297) (Conigrave and Hampson, 2006; Geng et al., 2016). Among these residues, E297 was identified through the natural mutation E297K as essential for structural and functional activity (Table 8) (Pollak et al., 1993; Bai et al., 1998; Conigrave et al., 2000; Zhang et al., 2002; Mun et al., 2004).

As recently identified NAMs, anions SO_4_^2−^ and PO_4_^3−^ are important modulators of the Ca^2+^ induced response. They bind in the VFT region and act as moderate NAMs for CaSR activity (Geng et al., 2016; Centeno et al., 2019). Based on anomalous difference maps, four anion-binding sites were identified in the inactive and active CaSR ECD structures. Sites 1 and 3 are located above the interdomain cleft in LBD1, while site 4 lies in the LBD2 region. Sites 1 and 3 appear to stabilize the inactive conformation while site 2, which is present in both active and inactive conformations appears important for receptor function as mutation in its residues (R66H, R69E, and S417L) abolished the Ca^2+^ induced response. In addition, each protomer structure contains one Ca^2+^ ion and three SO_4_^2−^ ions which together contribute to the structural integrity of the receptor (Geng et al., 2016). Taken together, anions along with Ca^2+^ and amino acids are involved in an intricate interplay for CaSR activation to maintain conformational equilibrium between inactive and active states.

As positive allosteric modulators, γ glutamyl peptides including glutathione (γGlu-Cys-Gly) and its analogs (Table 8) are predicted to have overlapping binding sites with L-amino acids in the VFT region (Wang et al., 2006; Ohsu et al., 2010; Broadhead et al., 2011). Kokumi peptides that activate CaSR resemble amino acids in having free α-amino and free α-carboxylate groups because they contain both amide bond formation between the γ-carboxylate group of L-glutamate and the α-amino group of its neighboring Cys residue. However, compared to amino acids, glutathione analogs have much larger side chains and are more potent activators of CaSR (Wang et al., 2006). Nonetheless, the free sulfhydryl is not required for CaSR activation (Ohsu et al., 2010; Maruyama et al., 2012).

The crystal structure of ECD enables mapping of the GSH binding site and investigation into how GSH binding works in synergy with Ca^2+^ to modulate the kokumi sensation. NPS2143, the sole kokumi NAM identified to date has been reported to inhibit kokumi taste sensation to GSH and its analogs which provides an opportunity to screen for novel kokumi enhancing molecules in a cell-based assay.

#### Synthetic Drugs as Allosteric Ligands of Calcium-Sensing Receptor

Because of its pathophysiological importance, various synthetic PAMs and NAMs of CaSR have been identified and are in clinical use. The allosteric modulation of CaSR by synthetic drugs has been recently reviewed (Hannan et al., 2016; Chavez-Abiega et al., 2020; Leach et al., 2020). Since the 1990’s the term calcimimetics and calcilytics, have been used for drugs that mimic or antagonize the effect of extracellular Ca^2+^ on CaSR activity, respectively. Pharmacologically, a calcimimetic activates the CaSR and includes agonists (type I) and allosteric ligands (type II). Most type I calcimimetics are either inorganic or organic polycations (e.g., Mg^2+^, Gd^3+^, neomycin), whereas type II calcimimetics are small naturally occurring molecules (aromatic amino acids or GSH) or synthetic drugs and peptides (NPS R-568, cinacalcet). Type II calcimimetics (like aromatic amino acids) bind in the ECD while others (e.g., NPS R-568, NPS R-467) bind in the TMD of the CaSR. Calcilytics are thus small organic molecules that appear to act as NAMs and bind in the TMD of the receptor (Widler, 2011; Nemeth, 2013).

Homology modeling and mutational studies show that both PAMs and NAMs have overlapping but non-identical binding sites in TMD and can partially allosterically modulate CaSR activity in the complete absence of the ECD, but their potencies vary among structurally different compounds (Collins et al., 1998; Ma et al., 2011) (Table 8). Several residues reportedly critical for allosteric modulation, W818^6.48^, F821^6.51^ (TMD6) and E837^7.39^, I841^7.43^ (TMD7), R680^3.28^, F684^3.32^, F688^3.36^ (TMD3) impair calcimimetic and calcilytic induced CaSR signaling (Miedlich et al., 2004; Petrel et al., 2004; Leach et al., 2016). Nevertheless, subtle differences in ligand–receptor interactions drive negative vs. positive modulation of CaSR signaling, by NPS2143 or cinacalcet and NPSR-568, respectively (Miedlich et al., 2004; Leach et al., 2016; Keller et al., 2018). The details of CaSR allosteric modulation by synthetic drugs is out of the scope of the current review, for a comprehensive explanation refer to these studies (Chaves-López et al., 2014; Hannan et al., 2016; Leach et al., 2020).

## Conclusion

Taste GPCR research has advanced rapidly over the past two decades providing a more thorough understanding of receptor molecular pharmacology and signal transduction pathways. With the exception of the kokumi receptor ECD, high-resolution crystal structures for any taste receptor would be a major step toward designing novel and potent surrogate taste receptor ligands and selective antagonists. This has been a challenge due to low taste GPCR functional heterologous expression, appropriate post-translational modifications, high conformational flexibility, and low detergent stability. However, significant advancements in structural biology technologies of serial femtosecond crystallography using X-ray free-electron lasers and high-resolution cryo-electron microscopy provide promising tools for understanding conformational dynamics and visualizing the process of receptor activation with high spatial and temporal resolution. The physiological relevance of taste GPCRs will be further advanced through *in vivo* studies to help provide information on potential synergies in taste signal transduction mechanisms particularly among bitter, umami, sweet and kokumi receptors.

## Author Contributions

RA wrote the manuscript and prepared the figures and tables; JD contributed to writing and reviewing the manuscript.

## Funding

This work was supported by an Endeavour Research Programme grant entitled “Accelerated evolution: A step change in food fermentation” (C10X1707) from the Ministry of Business Innovation & Employment, New Zealand.

## Conflict of Interest

The authors declare that the research was conducted in the absence of any commercial or financial relationships that could be construed as a potential conflict of interest.
