# Supplementary material for: G Protein-Coupled Receptors in Taste Physiology and Pharmacology
Source: Front Pharmacol. 2020 Nov 30;11:587664. doi: 10.3389/fphar.2020.587664 (PMC7774309; doi:10.3389/fphar.2020.587664)
Supplement: Supplementary file 1 [file table1.pdf]

Supplementary table 1. Bitter receptors and their ligands with their half maximal effective conc. (EC50) values

**Agonists**											**hTAS2R (mM)**										**Ref.**
**Receptors**	**1**	**3**	**4**	**5**	**7**	**8**	**9**	**10**	**13**	**14**	**16**	**38**	**39**	**40**	**43**	**44**	**46**	**47**	**49**	**50**	
Absinthin								0.1		0.1							0.001	0.0001			Cowart et al., 1994; Meyerhof et al., 2010; Talmon et al., 2019
Adhumulone	0.0001													0.00003							Intelmann et al., 2009
Aesculin											4										Bufe et al., 2002
Acesulfame K															4.7	0.34					Meyerhof et al., 2010
Acetaminophen													3								Rodgers et al., 2006;Dotson, et al,2008;Meyerhof et al., 2010
Acetylgenistin													0.125								Roland et al., 2013
Acetylthiourea												0.003									Meyerhof et al., 2010
Adlupulone	0.001									0.003											Intelmann et al., 2009
Aloin															0.0003	0.03					Meyerhof et al., 2010
Allylisothiocyanate												0.01									Meyerhof et al., 2010
Amarogentin	0.03		0.3										0.3		0.03		0.01	0.003		0.1	Glendinning, 1994;Meyer

																					hof et al., 2010
Amygdalin, D											3.2										Meyerhof et al., 2010
Andrographolide																	0.003	0.03		0.01	Meyerhof et al., 2010
Apigenin										0.008			0.001								Roland et al., 2011
Arborescin	0.1		0.03					0.1		0.1					0.1		0.003				Meyerhof et al., 2010
Arbutin											0.7										Meyerhof et al., 2010; Sakurai et al., 210; Bufe et al., 2002
Arglabin								0.1		0.1					0.003		0.003				Meyerhof et al., 2010
Aristolochic acid															1.1	0.00002					Meyerhof et al., 2010
Artemorin			0.1					0.003		0.003							0.01	0.03			Meyerhof et al., 2010
Azathioprine			0.3					0.3					1				0.3				Meyerhof et al., 2010
Benzamide										0.3											Meyerhof et al., 2010
Benzoin								0.03		0.01											Meyerhof et al., 2010
Biochanin Ag										0.063			0.5								Roland et al., 2013
Brucine								0.1									0.01				Meyerhof et al., 2010; Mattes, 1994
Butein										0.016			0.125								Roland et al., 2013

Caffeine					0.3			0.3		0.3					0.3		0.3				Burdock, 2005; Rodgers et al., 2006; Intelmann et al., 2009; Guinard et al., 1994
Chrysin										0.063			0.016								Roland et al., 2011
D-camphor			0.3					0.3		0.003								0.3			Meyerhof et al., 2010; Burdock., 2005
Caprolactam												10									Meyerhof et al., 2010
Carisoprodol										0.1							0.01				Meyerhof et al., 2010
Cascarillin	0.1							0.1		0.1							0.01	0.1			Meyerhof et al., 2010
Chalcone										0.032											Roland et al., 2011
Chloramphenicol	0.1					0.03		0.1					1		0.1		0.01				Meyerhof et al., 2010
Chlorhexidine										0.0001											Meyerhof et al., 2010
Chlorophenira mine			0.03		0.01			0.01		0.1		0.1	0.1	0.1			0.1				Meyerhof et al., 2010
Chloroquine		0.01						10		0.01			0.1								Meyerhof et al., 2010
Clonixin										0.002											Levit et al., 2014
Cnicin																	0.003				Meyerhof et al., 2010
Colchicine			0.1										3				0.3				Meyerhof et al., 2010
Colupulone	0.0001													0.00003							Intelmann et al., 2009

Coumarin								0.3		0.3											Burdock, 2005; Meyerhof et al., 2010
Coumestrol										0.25			0.25								Roland et al., 2011
Crispolide																	0.01				Meyerhof et al., 2010
Cromolyn					3										3						Meyerhof et al., 2010
Cucurbitacin B								0.00001		0.1											
Cucurbitacin E								0.00001													Meyerhof et al., 2010
Cycloheximide								0.1													Meyerhof et al., 2010
Cyanidine chloride										0.25			0.032								Roland et al., 2013
Dapsone			0.1					0.1						0.03							Meyerhof et al., 2010
Datiscetinb										0.002			0.016								Roland et al., 2013
Denatonium benzoate			0.3			1		0.003	0.03				0.1		0.3		0.03	0.00003			Meyerhof et al., 2010
Dextromethorphan	0.01							0.01													Meyerhof et al., 2010
Diclofenac										0.025											Levit et al., 2014; Schiffman et al., 2000
5,4'-dihydroxyflavone										0.25			0.5								Roland et al., 2013
5,7'-dimethoxyflavonee										0.016			0.032								Roland et al., 2013
Dimethyl-thioformamide												0.01									Meyerhof et al., 2010

Diphenhydramine										0.03				0.03							Meyerhof et al., 2010
Diphenidol	0.1		0.1		0.01			0.03	0.03	0.01	0.1	0.1	0.1	0.03	0.03	0.003	0.03	0.1	0.1		Meyerhof et al., 2010
Diphenylthiourea										0.0029		0.0003									Meyerhof et al., 2010; Dotson et al., 2008
3,5-Diiodosalicylic Acid										0.0005											Levit et al., 2014
Divinylsulfoxide										3											Meyerhof et al., 2010
Dulcoside A			0.2							0.05											Hellfritsch et al., 2012
(-)-Epicatechin, EC			2	1									1								Drewnowski & Gomez-Carneros,2000
Epigallocatechin gallate, egcg													0.01		0.01						Drewnowski & Gomez-Carneros,2000
(+/-) Equol										0.008			0.032								Roland et al., 2011
Eriodictyol										0.032			0.016								Roland et al., 2013
Erythromycin								0.3													Dotson, et al, 2008;Meyerhof et al., 2010
Ethylpyrazine												0.3									Meyerhof et al., 2010; Dotson et al., 2008
N-ethyltiourea												0.03									Meyerhof et al., 2010

N,N´-ethylene thiourea																					
Falcarindiol										0.1					0.1						Meyerhof et al., 2010
Famotidine								0.3								0.3					Meyerhof et al., 2010
Fisetin													0.001								Roland et al., 2013
Formomonetin										0.5			0.5								Roland et al., 2013
Flavone										0.008			0.008								Roland et al., 2013
Flavanone										0.032											Roland et al., 2013
Flufenamic acid										0.00001											Meyerhof et al., 2010; Di Pizio et al., 2020
Fustin										0.5			0.25								Roland et al., 2013
Genistin										0.004			0.25								Roland et al., 2011; Drewnowski & Gomez-Carneros,2000
Genkwanin													0.5								Roland et al., 2013
Gossypetin													0.25								Roland et al., 2013
Grosshemin															0.1		0.001				Meyerhof et al., 2010
Glycitein										0.5			0.5								Roland et al., 2013
Gly-leu	100																				Maehashi et al.,2008
Gly-phe	57.5																				Maehashi et al.,2008;

																					Upadhyaya et al., 2010
Haloperidol								0.03		0.03											Meyerhof et al., 2010
Herbacetin										0.125			0.125								Roland et al., 2013
Herbolide D																	0.01				Brockhoff et al., 2007
Helicin											0.4				1						Meyerhof et al., 2010; Bufe et al., 2002
Hesperitin										0.016			0.008								Roland et al., 2013
Hg-12					0.25					0.25											Le Neve et al., 2010
Homoeriodictyol										0.032			0.032								Roland et al., 2013
Humulon	0.0001													0.0001							Intelmann et al., 2009
Hydrocortisone																	0.003				Meyerhof et al., 2010
4-hydroxyanisol										0.3											Meyerhof et al., 2010
5-Hydroxyflavone													0.5								Roland et al., 2013
Cis-isoadhumulone	0.0003									0.001											Intelmann et al., 2009
Isoflavone										0.25											Roland et al., 2013
Isorhamnetin										0.125											Roland et al., 2013
Isoxanthohumol	0.003									0.003				0.01							Intelmann et al., 2009

Cis-isocohumulone	0.001									0.001											Intelmann et al., 2009
Cis-isohumulone	0.0003									0.0003											Intelmann et al., 2009
Kaempherol										0.008			0.014								Roland et al., 2013; Levit et al., 2014
Limonin												0.1									Meyerhof et al., 2010; Drewnowski, & Gomez-Carneros, 2000; Guinard et al., 1994
Liquiritigenin										0.032			0.016								Roland et al., 2013
Leu-trp	3		3										3								Kohl et al., 2013
Leu-leu-leu			3																		Kohl et al., 2013
Liquiritigenin										0.032			0.016								Roland et al., 2013
Lupulon	0.0001									0.003											Intelmann et al., 2009
Luteolin										0.002											Roland et al., 2013
Malathion										0.05											Levit et al., 2014
Mefenamic acid										0.003											Levit et al., 2014; Ayenew et al., 2009; Alshehri et al., 2015
Methimazole												0.03									Meyerhof et al., 2010

4(6)-methyl-2 thiouracil												0.03									Meyerhof et al., 2010
Methyl beta-D-glucoside											15										Bufe et al., 2002
Methylthiourea												0.1									Dotson et al., 2008; Meyerhof et al., 2010
Miconazole										0.033											Levit et al., 2014
Morin										0.008			0.002								Roland et al., 2013
Myricetin										0.25			0.001								Roland et al., 2013
1-Naphthoic acid										0.003											Behrens et al., 2004
2-Naphthyl beta-D-glucopyranoside											0.4										Bufe et al., 2002
2-nitro phenyl beta-D-glucopyranoside											1.5										Bufe et al., 2002
6-Nitrosaccharin												0.1									Hamor, 1961
Noscapine										0.01											Meyerhof et al., 2010
Ofloxacin							0.2														Dotson et al., 2008
Orphenadrine																	0.03				Meyerhof et al., 2010
Pantoprazole										0.1											Levit et al., 2014
Papaverine					0.01			0.01		0.01											Meyerhof et al., 2010; Guinard et al., 1994

Pemirolast										0.005											Levit et al., 2014
Parthenolide	0.1		0.03			0.1		0.03		0.003						0.1	0.001				Meyerhof et al., 2010
Pelargonidin chloride										0.063			0.032								Roland et al., 2013
Pentagalloylglucose (PGG)				0.003									0.003								Soares et al., 2013
Phloretin										0.016			0.008								Roland et al., 2013
Picrotin										0.03											Behrens et al., 2004
Picrotoxinin	1							1		0.003							0.01	1			Meyerhof et al., 2010
Pinocembrin										0.008			0.004								Roland et al., 2013
Piperonylic acid										0.1											Behrens et al., 2004
1,10-phenanthroline				0.1																	Meyerhof et al., 2010
Phenyl beta-D-glucopyranoside											0.38										Sakurai et al., 2010; Bufe et al., 2002
Phe-leu	7.2 (EC50)																				Upadhyaya et al., 2010
L-phenylalanine	53.3																				Kohl et al., 2013;Burdock, 2005
Phe-trp	1		1																		Kohl et al., 2013
Phe-phe-phe	0.37 (EC50)																				Upadhyaya et al., 2010
phenylethyl isothiocyanate												0.03									Meyerhof et al., 2010
Phenylthiocarbamide (PTC)												0.00004									Dotson et al., 2008; Meyerhof et al., 2010

Pirenzapin							1.8														Dotson et al., 2008
8-prenylnaringenin										0.0003											Intelmann et al., 2009
Procyanidin C2				0.03																	Soares et al., 2013
Pro-arg													10								Ueno et al., 2011
Procainamid							2.8														Dotson et al., 2008
Propylthiouracil (PROP)												0.011									Dotson et al., 2008; Meyerhof et al., 2010
Quassin			0.3					0.3		0.3							0.3	0.3			Rodgers et al., 2006; Dotson et al., 2008; Meyerhof et al., 2010
Quercetin										0.001											Drewnowski, & Gomez-Carneros, 2000
Quinine			0.01		0.01			0.01		0.01			0.01	0.01	0.01	0.01	0.01				Meyerhof et al., 2010
Resveratrol										0.016			0.063								Roland et al., 2013
Saccharin															0.28	0.14					Rodgers et al., 2006; Meyerhof et al., 2010
Strychnine								0.003									0.0001				Meyerhof et al., 2010
Naringenin										0.01											Fuhr et al., 1993
Niflumic acid										0.005											Levit et al., 2014

Rebaudioside A			0.2							0.6											Hellfritsch et al., 2012
Rebaudioside B			0.2							1											Hellfritsch et al., 2012
Rebaudioside C			0.4							0.4											Hellfritsch et al., 2012
Rubusoside			0.05																		Hellfritsch et al., 2012
Scutellarein										0.008			0.008								Roland et al., 2013
(−)-α-Santonin								0.1		0.1							0.1				Lossow et al., 2016
D-Salicin											0.09										Rodgers et al., 2006; Dotson et al., 2008; Intelmann et al., 2009; Bufe et al., 2002
Salsalate										0.1											Levit et al., 2014
Silibinin										0.008			0.008								Roland et al., 2013
Sinigrin											0.1	0.1									Meyerhof et al., 2010
Sintenin																	0.00 03				Brockhoff et al., 2007
Sodium benzoate										0.3	3										Meyerhof et al., 2010
Sodium cyclamate	30											30									Meyerhof et al., 2010
sodium thiocyanate	3											1									Meyerhof et al., 2010
Stevioside			0.2																		Hellfritsch et al., 2012
Steviolbioside			0.4																		Hellfritsch et al., 201

Strychnine								0.003									0.0001				Meyerhof et al., 2010
Sucrose Octaacetate																	0.3				Burdock, 2005; Meyerhof et al., 2010
Sulfuretin										0.016			0.016								Roland et al., 2013
Tatridin A																	0.03				Brockhoff et al., 2007
Tatridin B																	0.1				Brockhoff et al., 2007; Meyerhof et al., 2010
(+)- Taxifolin										0.063			0.125								Roland et al., 2013; Intelmann et al., 2009
Teuflavin																	0.03				Brockhoff et al., 2007
Thiamine	1												1								Meyerhof et al., 2010
Theobromine										1											Burdock, 2005; Guinard et al., 1994
Trans-isoadhumulone	0.0003									0.001											Intelmann et al., 2009
Trans-isocohumulone	0.001									0.001											Intelmann et al., 2009
Trans-isohumulone	0.0003									0.001											Intelmann et al., 2009
L-tryptophan			10																		Kohl et al., 2013;Sonntag et al., 2010

Trp-leu			3																		Kohl et al., 2013
Trp-phe			0.3																		Kohl et al., 2013
Trp-pro			3																		Kohl et al., 2013
Trp-trp-trp	0.1		0.01							0.1			0.1				0.1				Kohl et al., 2013
Trp-trp	0.3		1										1								Kohl et al., 2013
Thujone, (-)-α-								0.1		0.003											Meyerhof et al., 2010
Tricetin													0.25								Roland et al., 2013
Umbelliferones										0.6											Rodgers et al., 2006
Vulgarolide																	0.1				Brockhoff et al., 2007
Xanthohumol	0.001									0.003				0.003							Intelmann et al., 2009
Xanthone										0.25			0.5								Roland et al., 2013
Xanthotoxin								0.1		0.1							0.1				Mancuso et al., 2015
Yohimbine	0.3		0.3					0.3				0.3					0.3				Meyerhof et al., 2010
